# Adaptive information-constrained mapping for feature compression in edge AI and federated systems

**DOI:** 10.1038/s41598-025-16604-2

**Published:** 2025-08-22

**Authors:** Viacheslav Kovtun

**Affiliations:** 1 Vinnytsia National Technical University, Vinnytsia, Ukraine; 2 Institute of Theoretical and Applied Informatics, Polish Academy of Sciences, Bałtycka 5, 44-100 Gliwice, Poland

**Keywords:** Engineering, Mathematics and computing

## Abstract

This article explores the problem of efficient feature compression in distributed intelligent systems with limited resources, particularly within the context of Edge AI and Federated Learning. The relevance of this study is driven by the growing need to reduce communication overhead under conditions of unstable Quality of Service, limited bandwidth, and high heterogeneity of input data. The scientific novelty lies in the development of a consistent entropy-regularised compression model that combines variational latent mapping, non-negativity-constrained projection design, and stochastic-Boolean transformation of the feature space. A generalised compression quality functional is proposed, integrating the directed Kullback–Leibler divergence, an entropic regularisation component, and a guarantee of preserving the semantic relevance of the compressed representation. Efficient projection-gradient optimisation algorithms have been developed, suitable for implementation in constrained computational environments. The practical effectiveness of the approach has been confirmed through experiments on the HAR and PAMAP2 datasets: a 6–eightfold reduction in entropy load was achieved while maintaining classification accuracy above 94% and a high level of semantic fidelity in the reconstructed data. The models were deployed on low-power devices (Jetson Nano, Raspberry Pi 4), where they demonstrated robustness to noise and loss, as well as superiority over current SOTA solutions (FedEntropy, EDS-FL, SER) in terms of compression efficiency, adaptability to heterogeneous distributions, and stability under unstable transmission conditions.

## Introduction

### Relevance of the Research

Modern information processes are increasingly implemented under conditions of distribution, limited computational resources, unstable communication channels, and high variability of input data. This is typical of a wide range of applied systems: wearable devices, autonomous sensor networks, mobile IoT platforms, urban infrastructures, industrial facilities, and distributed energy grids. In such environments, the tasks of feature collection, transmission, and processing require not only efficiency but also robustness to external and internal disturbances.

In the context of tasks such as human physical activity monitoring^1^, equipment condition diagnostics^2^, user behaviour analysis^3^, or local energy system stability control^4^, there arises a need to reduce the volume of information circulating within the system. However, data reduction must not result in the loss of its practical value. The concern lies not merely in the technical transmission of values but in preserving the portion of information that is critical for subsequent analysis, classification, prediction, or control. It is this applied essence, namely the semantic significance of features, that must remain intact under any transformations. Implementing compression under such conditions presents several challenges. Incoming data streams are often redundant, noisy, or incomplete. This complicates the preservation of their informative structure after compression. Transmission channels are characterised by instability, variable bandwidth, and intermittency, which increases the risk of loss. Moreover, the hardware components of edge devices are subject to significant limitations in terms of memory capacity, energy consumption, and computational power, rendering the use of full-scale classical analysis methods unfeasible without prior feature reduction.

Most existing data compression approaches focus primarily on volume reduction, without accounting for the need to adapt to the applied context^5^. They do not provide flexible control over which information is preserved and which is discarded, often resulting in significant efficiency losses at subsequent processing stages. In view of this limitation, our research is focused on formalising approaches to semantically controlled feature compression capable of operating in complex information environments. Particular attention is given to the adaptation of such methods to hardware-constrained systems, the preservation of the functional relevance of features, and the assurance of robustness to the variability of data received from sensor sources. Therefore, the core problem addressed in this work is how to design an information-constrained feature encoding mechanism that simultaneously achieves bit-level compactness, task-specific relevance, and semantic preservation, without relying on post hoc selection or handcrafted compression pipelines.

## State-of-the-art

In recent scientific literature, a number of approaches have emerged aiming to reduce information load in applied systems without compromising the functional informativeness of features. These approaches differ significantly in their underlying concepts, architectural implementation, and application domains () ranging from lightweight models for local inference on low-power devices to stochastically regularised autoencoders and communication-efficient schemes designed for distributed processing. A critical analysis will be conducted of the most relevant directions that currently constitute the core of modern solutions in the field of semantically controlled feature compression within distributed information systems.

Semantically controlled feature compression^6,7^ involves reducing the dimensionality or complexity of input representations while preserving the information that is critical for performing a target task (classification, regression, anomaly detection, etc.). In contrast to traditional compression approaches, which focus on global reconstruction metrics, this class of methods incorporates importance features directly into the training process—via gradient-based maps (e.g. Grad-CAM, LRP)^8^, attention mechanisms^9^, or task-aware regularisation^10^. Notable implementations include supervised bottleneck architectures^11^, semantic dropout^12^, and feature masking with learnable importance weights^13^. For instance, studies^14,15^ have demonstrated the effectiveness of attention-guided compression in mobile video stream classification and biomedical signal processing tasks. However, these models have several limitations: they typically require annotations or feedback from the main task, which complicates deployment under limited supervision; in addition, integrated attention modules increase the number of parameters, which contradicts the requirements for lightweight systems in edge environments. In some cases, there is also sensitivity to noise in semantic maps, which reduces the model’s robustness during inference under unstable conditions.

Entropy-constrained representation learning^16^ is based on controlling the information capacity of the latent space through specialised regularisers that limit the volume of transmitted or retained information. Classical implementations take the form of variational autoencoders (VAE) with modified loss functions incorporating terms such as KL divergence or mutual information bounds (e.g. β-VAE, InfoVAE, δ-VAE)^17,18^. These models enable the formation of a compact yet expressive latent space suitable for subsequent use in classification or reconstruction tasks. Additionally, learnable entropy models^19^ are employed to parameterise entropy via scalable coders. In image recognition and audio compression tasks, these approaches have demonstrated effective bandwidth reduction without compromising accuracy. However, the primary drawback of such methods lies in their training complexity: optimisation involving entropy regularisers often leads to unstable convergence and high sensitivity to the choice of hyperparameters ($\beta$, $\lambda$). Furthermore, the resulting latent spaces may exhibit low separability or complex topology, complicating interpretation and subsequent use in low-power inference. In certain cases, a trade-off is also observed between entropy efficiency and the semantic relevance of features.

Task-oriented feature reduction involves selecting or transforming only those components of the input space that are most informative for a specific task (such as action classification, state prediction, or anomaly detection). Unlike universal compression methods, these approaches incorporate knowledge of the target function directly into the model training process. The most common techniques include differentiable feature masking mechanisms (learnable masks)^20^, attention-based selection^21^, and joint learning models with sparsity control blocks (e.g. L0 regularisation, concrete masks, reinforcement-driven feature gating). Applied implementations include the Adaptive Feature Selection Network (AFSNet)^22^, Differentiable Feature Selection (DFS)^23^, and two-stage selection methods in which the reducer is trained separately from the predictor. The key advantage of such approaches lies in their flexibility: they enable a reduction in the number of features without loss of performance and allow adaptation to changing conditions or domains. However, major drawbacks remain: high sensitivity to distributional shifts, reliance on full access to labelled examples, and challenges in generalising to new tasks under weak supervision. Moreover, many existing solutions overlook the entropic complexity of residual features, which complicates their subsequent transmission over constrained channels.

Stochastic and discrete latent spaces^24^ are employed to construct generalised and compact representations that are robust to noise and partial loss of input features. This approach models the latent space as a random or quantised variable with controlled variance, enabling the avoidance of overfitting to overly deterministic structures and enhancing the generative capacity of models. One classical example is the reparameterisation trick in VAEs, while more recent developments include discrete encoding in VQ-VAE, Gumbel-softmax quantisation, as well as Concrete Bernoulli and Binary Concrete models. These methods are widely used in low-rate compression, representation learning under unsupervised conditions, and tasks involving corrupted or partially missing input. Their advantage lies in the ability to retain structural informativeness even under unstable input streams. However, their drawbacks include training complexity due to the non-differentiability of quantisation operations (requiring additional approximations), the risk of generating “dead” codes in discrete dictionaries, and limited interpretability of latent features. Moreover, such models require careful tuning of temperature and regularisation parameters, which complicates their deployment in dynamic edge analytics environments.

Communication-efficient encoding in the context of federated learning (FL) and IoT systems focuses on minimising the volume of transmitted features or parameters while preserving the applied accuracy of the model^25,26^. This approach is critically important in scenarios where communication channels are narrowband, unstable, or energy-expensive, such as in mobile sensor networks or edge clouds. Key methods include sparsification strategies (e.g. top-*k* selection, random-$k$ dropping), gradient quantisation (QSGD, TernGrad), adaptive update scaling (AdaComp), and split learning with model partitioning between device and server. At the feature level, importance-aware encoding^27^ and rate-aware communication schemes^25^ are being actively developed to adjust transmission volume according to the entropy budget. These solutions enable substantial traffic reduction without significant accuracy loss, particularly in large-scale client networks. However, they also face several limitations: the effectiveness of many approaches heavily depends on client synchrony and channel stability, which is not always feasible in real-world IoT environments; some algorithms require prior knowledge of input feature statistics; moreover, aggregation and selection are often performed using heuristic rules, which limits generalisability and adaptability when the task or domain changes.

Despite significant progress in the development of feature compression approaches in distributed systems, none of the examined directions ensures the simultaneous achievement of high semantic relevance, entropy efficiency, task adaptability, and robustness to unstable transmission conditions. Semantically controlled methods require annotations and increase computational complexity; entropy-constrained learning suffers from difficult optimisation and instability of the latent space; task-oriented reduction is sensitive to distribution shifts and often neglects communication constraints; stochastic and discrete spaces pose training challenges and offer limited interpretability; communication-efficient encoding, in turn, is largely heuristic-based, limiting its generalisability. All this highlights the need for a comprehensive approach to feature compression that combines the advantages of these directions while overcoming their key shortcomings. In this context, it is reasonable to develop an entropy-regularised feature transformation system capable of adaptively aligning compression with the applied significance of features and the parameters of the transmission environment.

In this context, it is appropriate to turn to modern state-of-the-art (SOTA) methods that implement the most promising approaches to feature compression in federated and distributed learning. Three of them deserve particular attention as the closest to the proposed model in terms of target orientation: FedEntropy, EDS-FL, and SER. FedEntropy^28^ is based on the principle of entropy-constrained quantisation aimed at improving communication efficiency in federated learning. The method incorporates an entropy regulariser into the loss function, enabling the reduction of redundancy in transmitted features without significant loss of informativeness. It adapts the encoding precision to the local entropy profile, but is heavily dependent on the encoder configuration and shows reduced generalisability to new classes or under heterogeneous data distributions. EDS-FL^29^ is a task-centric compression strategy implemented via knowledge distillation. It involves training a compact representation for each client under the guidance of a shared teacher model, allowing task-relevant characteristics to be preserved and variability to be reduced. This approach is effective under stable conditions with a fixed number of classes but loses performance when scaled to a larger number of clients or changing tasks. An additional challenge is the need for synchronised training and increased computational overhead. SER^30^ is a stochastic encoding and reconstruction framework that uses probabilistic latent representations to model uncertainty in features. Compression is achieved by sampling from a trained distributional model, ensuring high compression while maintaining representativeness. However, such stochasticity is accompanied by optimisation difficulties, high variability, and low interpretability of the latent space, complicating alignment with the structure of target tasks. Each of these methods illustrates an important aspect of the feature compression problem in distributed systems—entropy, task-centricity, or latent flexibility. Yet none offers a comprehensive solution combining semantic relevance, entropy efficiency, task adaptability, and robustness to transmission constraints. A comparative analysis of the main characteristics of these methods and the proposed solution is presented in Table 1.

**Table 1:** Conceptual comparison of the proposed method and three representative SOTA approaches.

Criterion	Proposed (Ours)	FedEntropy	EDS-FL	SER
Design paradigm	Regularised representation optimisation	Entropy-constrained quantisation	Task-oriented distillation	Stochastic latent encoding
Entropy reduction potential	High (explicit regularisation of representation entropy)	Moderate (adaptive to local entropy)	Moderate (guided reduction via distillation)	Low (uncontrolled stochastic variability)
Semantic fidelity	High (controlled latent structure)	Moderate (depends on encoder configuration)	Moderate (teacher-guided relevance)	Low (sample-level uncertainty dominates)
Task adaptivity	High (task-aware training objectives)	Moderate (manual hyperparameter tuning)	Moderate (aligned via teacher)	Low (static latent sampling)
Generalisation capability	High (validated for unseen classes/tasks)	Low (encoder-specific generalisation)	Moderate (task-bound distillation)	Low (limited transferability)
Compression adaptability	High (context-aware encoding)	Moderate (entropy-profile driven)	Moderate (fixed distillation targets)	High (sampling enables variability, less control)
Latent space consistency	High (stabilised via regularisation)	Moderate	Low (inter-client feature divergence)	Low (sampling noise)
Interpretability	Moderate–High (structured latent space)	Low	Moderate	Low
Scalability	High (50 + clients, multiple tasks supported)	Moderate (tested on small-scale setups)	Low (distillation overhead)	Moderate (client-specific tuning needed)
Communication efficiency	High (learned, compact, and robust encoding)	Moderate (entropy heuristics)	Low (additional teacher–client exchange)	Moderate (bit-level control, low robustness)
Training complexity	Moderate (no external modules)	Moderate	High (teacher synchronisation required)	Moderate–High (training instability)
Robustness to data heterogeneity	High (validated under non-IID distributions)	Moderate (entropy adapts partially)	Low (sensitive to class imbalance)	Low (amplified by stochasticity)
Inference suitability for edge devices	High (low latency and model size)	Moderate	Low (teacher model size dominates)	Moderate (requires sampling overhead)

Although numerous recent studies have addressed aspects of feature compression and efficiency in federated and distributed environments, several gaps remain that motivate the present research. For instance, the approach in^31^ proposes an autoencoder-based federated learning scheme for constrained IoT devices, but it lacks entropy-level control and exhibits limited adaptability to heterogeneous data distributions. Similarly, while^32^ explores compressive techniques under noise and imbalance, it predominantly relies on heuristic sampling strategies and omits compactness at the bit level. In contrast,^33^ presents an energy-aware FL scheme optimised for client-side deployment, yet the encoder-decoder structure requires high-precision floating-point operations, restricting its applicability in low-power hardware. Furthermore, the method introduced in^34^ integrates blockchain for secure edge learning but does not address the information bottleneck problem or compression trade-offs. Although^35^ leverages ensemble pruning techniques to reduce overhead, it does not preserve latent space topology or feature-level relevance, which is essential for semantically aware representations. Moreover, the reinforcement-based approaches in^36^ and^37^ focus on dynamic task selection and attention-based encoding respectively, achieving moderate accuracy improvements but suffering from convergence issues and excessive memory footprints in embedded contexts. While^38^ utilises hybrid FL schemes for video-based applications, it is not applicable to low-latency sensory environments. In^39^, entropy-regularised FL is proposed, yet it depends heavily on centralised tuning parameters and fails to maintain performance across variable client setups. Additionally, the studies in [,^40,41^ either apply classical clustering, probabilistic neural representations, or watermarking for privacy, but none of them provide a unified mechanism to ensure compression ratio, semantic retention, and client-specific adaptation simultaneously. While each of the reviewed approaches contributes to various facets of efficient FL and encoding, their limitations highlight the need for an integrated mechanism that jointly ensures task relevance, semantic fidelity, and computational tractability at bit-level precision. Therefore, building upon the identified gaps, the present work proposes an adaptive, information-constrained feature encoding scheme that unifies variational and stochastic-Boolean compression under federated conditions with minimal reliance on post hoc filtering or handcrafted pipelines.

In addition to the methodological limitations identified above, recent applied studies further underscore the real-world demand for effective information-constrained feature compression across critical domains. For example, in the context of autonomous vehicles,^42^ applies hybrid convolutional frameworks for traffic sign recognition under bandwidth constraints, yet lacks bit-level compression or entropy control. Similarly,^43^ uses vision-based structural health monitoring, highlighting the need for compact, task-relevant embeddings in latency-sensitive edge deployments. In the IoT security domain,^44^ and^45^ explore intrusion detection and feature selection using LSTM-based and statistical techniques respectively, but these approaches rely heavily on centralised architectures and do not incorporate semantic-preserving compression. Likewise,^46^ and^47^ focus on energy prediction for smart buildings, where efficient feature representation plays a pivotal role in inference speed and transmission efficiency, though entropy regularisation is not addressed. In healthcare,^48^ present deep learning-based solutions for Alzheimer’s detection and elderly care respectively, yet these models typically involve dense parameterisations unsuitable for bandwidth-limited or privacy-sensitive environments. Furthermore,^49^ proposes a deep model for earthquake damage prediction, again without considering adaptive feature compression or decentralised inference. While these studies collectively affirm the pressing relevance of efficient and semantically aware representations in real-world applications, they do not offer an integrated, entropy-constrained compression mechanism applicable across heterogeneous deployment contexts. The present work aims to address this unmet need.

Despite notable progress in entropy-aware compression and task-oriented feature selection, existing solutions often treat these dimensions independently or rely on rigid, non-adaptive mechanisms. To the best of my knowledge, no prior work offers a unified framework that jointly optimises entropy, semantic fidelity, and deployment resilience under communication constraints. This methodological gap motivates the development of the proposed adaptive information-constrained mapping approach detailed in Sect. “Models and methods”.

## Main attributes of the research

The objective of this study is the process of entropy-constrained feature compression and reconstruction in resource-limited distributed intelligent systems, which involves stochastic-regularised transformation of the feature space to preserve semantic informativeness during data transmission and recovery.

The subject of this study comprises variational-entropy methods for constructing information-constrained representations of the feature space, in particular projection-gradient optimisation strategies and stochastic-regularised binary projectors, which enable analytical modelling of the trade-off between reconstruction accuracy and entropy compactness and formed the basis for the theoretical conclusions and scientific contribution of the research.

The aim of this study is to enhance the efficiency of feature compression in distributed intelligent systems, particularly in Edge AI and Federated Learning environments, by developing entropy-regularised methods for feature space transformation that reduce information loss, improve the semantic consistency of reconstruction, and ensure adaptability to constraints in bandwidth, energy consumption, and fragmentation of input data.

To achieve the stated aim, the following key research objectives were defined:

1. To develop a variational model of entropy-constrained feature compression that enables sequential formation of a latent representation, its selection, and reconstruction while preserving semantic consistency.
2. To formulate a generalised compression quality functional that integrates information divergence and entropy capacity criteria, and to define a minimisation strategy under orthant constraints.
3. To design a stochastic-regularised approach to Boolean projection that enables entropy-controlled compression in environments with limited bit-depth.
4. To construct computational algorithms for the implementation of both approaches, adapted to resource-constrained Edge AI architectures and capable of operating under unstable QoS conditions.
5. To conduct experimental evaluation of the trade-off between reconstruction accuracy and compression rate, as well as the impact of entropy regularisation parameters on recovery quality.
6. To perform a comparative analysis of the proposed methods against current SOTA solutions, considering classification accuracy, entropy efficiency, computational complexity, and adaptability to heterogeneous data.

The main scientific contributions of this study are as follows:

1. A unified entropy-regularised feature compression model is proposed, combining variational optimisation with orthant-constrained projection and a stochastic-Boolean transformation scheme. Unlike existing SOTA solutions (EDS-FL, FedEntropy, SER), this model ensures the preservation of semantic informativeness under constraints on bandwidth, energy consumption, and bit-depth.
2. A novel compression quality functional is formulated, integrating directed Kullback–Leibler divergence and entropy perturbation into a single optimisation criterion. Efficient projection-gradient algorithms have been developed for both continuous and discrete (Boolean) representations, enabling adaptive compression under conditions of heterogeneity, noise, and unstable QoS.
3. Empirical testing on the HAR and PAMAP2 datasets confirmed the practical effectiveness of the approach in edge/federated environments. The proposed methods achieve high classification accuracy while reducing entropy load by up to eightfold, demonstrating stable performance on low-power devices (Jetson Nano, Raspberry Pi 4), robustness to noise and data loss, and superiority over contemporary counterparts in terms of reconstruction accuracy and resource efficiency.

The structure of the article is organised as follows:

- Secting “Models and methods” presents the theoretical foundation and algorithmic principles of the proposed approach.
- Secting “Variational design of stochastic feature encoders” formulates a variational feature compression model based on a two-stage latent transformation followed by reconstruction under orthant constraints. A generalised compression quality functional is introduced, combining directed Kullback–Leibler divergence and entropy perturbation. A projection-gradient minimisation algorithm is proposed, designed for resource-constrained environments.
- Secting “Bit-level compression with entropy-driven selection” develops an alternative stochastic-Boolean strategy for entropy compression, based on the generation of binary projectors using Lagrangian regularisation. An analytical formula is derived for selecting the optimal configuration, taking into account entropy compactness and the information budget.
- Section “Results” details the experimental methodology. It describes the datasets used (UCI HAR, PAMAP2), procedures for generating heterogeneous non-IID distributions, simulating noise and partial feature loss. Hardware constraints on Jetson Nano and Raspberry Pi 4 devices are modelled. Baseline methods (EDS-FL, FedEntropy, SER) are presented, and a full set of metrics is defined: classification accuracy, entropy complexity variation, inference delay, memory load, and energy consumption.
- Sect. 4 presents the experimental results and their analysis. The ability of both proposed strategies to achieve a balance between reconstruction accuracy and the degree of feature compression is demonstrated. The effectiveness of the models under varying levels of entropy regularisation and the λ parameter is shown. Particular attention is given to the robustness of the proposed approaches to data heterogeneity, noise, and channel instability in Edge AI and Federated Learning scenarios.

## Models and methods

To enable effective feature compression under the constraints of decentralised environments, the proposed approach combines two complementary strategies: (i) a variational entropy-regularised projection method and (ii) a stochastic Boolean projection mechanism optimised for bit-limited transmission. The first strategy, detailed in Sect. “Variational design of stochastic feature encoders”, constructs a two-stage linear transformation and reconstruction pipeline governed by a variational optimisation functional that incorporates directed Kullback–Leibler divergence and entropy deviation. The second strategy, developed in Sect. “Bit-level compression with entropy-driven selection”, employs entropy-weighted selection over a finite set of binary projection matrices to generate compact representations without the need for backpropagation or gradient computation. This integrated design supports adaptive deployment across a variety of Edge AI and Federated Learning scenarios, accommodating diverse limitations in bandwidth, energy availability, and computational resources.

## Variational design of stochastic feature encoders

In many tasks involving distributed intelligent data processing, particularly in systems such as Edge AI and Federated Learning, the input information is received in the form of rectangular state matrices ${\text{X}} \in {\mathbb{R}}_{ + }^{n \times l}$, where $n$ denotes the number of local observations (e.g. sensor monitoring sessions or traffic packets), and $l$ represents the dimensionality of the original feature space. A typical example includes multichannel time series collected on resource-constrained end devices (smart sensors, IoT agents, or base stations such as eNB or gNB).

Given the constraints of bandwidth, energy budget, or privacy policy, it becomes necessary to transform ${\text{X}}$ into a reduced representation ${\text{Y}} \in {\mathbb{R}}_{ + }^{m \times k}$, $m < n$, $k < l$, suitable for further use in a global model or aggregation module. This transformation may involve a reduction in the number of instances $m$ as well as compression of feature dimensionality $k$, while preserving the informational essence of the data stream.

In a federated learning environment with data transmission over unstable channels (e.g. NB-IoT or URLLC), effective local compression requires the construction of a two-stage mapping aimed at preserving semantic completeness during compressed transmission. Formally, this is achieved through a sequential two-stage transformation.

In the first stage of direct information-constrained encoding, the input data matrix ${\text{X}}$ is mapped to an intermediate latent representation ${\text{U}} \in {\mathbb{R}}_{ + }^{n \times k}$, followed by transformation into a compressed format ${\text{Y}}$ according to procedure

1 $$\left\{ \begin{gathered} {\text{XR}} = {\text{U}}, \hfill \\ {\text{Q}}^{\left( \tau \right)} {\text{U}} = {\text{Y}}, \hfill \\ \end{gathered} \right.$$

where ${\text{R}} \in {\mathbb{R}}_{ + }^{l \times k}$ denotes the feature mapping matrix, and ${\text{Q}}^{\left( \tau \right)} \in {\mathbb{R}}_{ + }^{m \times n}$ is an adaptive selection matrix dependent on the parameter $\tau$, which characterises traffic priority or the current level of QoS.

The reverse reconstruction stage of the original structure, for example at a cloud aggregation node, is carried out in two steps:

2 $$\left\{ \begin{gathered} {\text{YB}} = {\text{C}}, \hfill \\ {\text{DC}} = {\text{A}}, \hfill \\ \end{gathered} \right.$$

where ${\text{B}} \in {\mathbb{R}}_{ + }^{l \times k}$ is the decoding matrix subject to a semantic deviation constraint, and ${\text{D}} \in {\mathbb{R}}_{ + }^{n \times m}$ is the spatial scale restoration operator. The intermediate result ${\text{C}} \in \mathbb{R}_{ + }^{{m \times l}}$ contains the reconstructed features for the selected subset of instances and acts as a latent informational core, from which the full-scale approximation ${\text{A}} \in {\mathbb{R}}_{ + }^{n \times l}$ is formed. This approximation is then used either during the global model generalisation phase or as input to the backward flow in the iterative optimisation cycle.

The projection operators ${\text{R}}$, ${\text{Q}}^{\left( \tau \right)}$, ${\text{B}}$, ${\text{D}}$ are integral components of the coherent mechanism for information-constrained compression and reconstruction. All these matrices are assumed to be ${\mathbb{R}}_{ + }$-defined, which aligns with the physical nature of features in the preprocessing of telemetry streams.

According to Eqs. (1) and (2), the reconstructed representation ${\text{A}}$ can be expressed as a composition of four interdependent transformations:

3 $${\text{A}} = {\text{D}}\left\{ {\left[ {{\text{Q}}^{\left( \tau \right)} \left( {{\text{XR}}} \right)} \right]{\text{B}}} \right\} > 0$$

Equation (3) generalises the full encoding–decoding cycle under conditions of limited resource availability: from local latent feature formation to their unfolding in the reconstructed space. The use of nested parentheses in (3) indicates a hierarchical sequence of operations: first, linearised feature compression; next, reduction of instance dimensionality according to the τ-dependent policy; and only then, feature recovery and spatial structure reconstruction. Accordingly, the transformation $\left( \cdot \right) \to \left[ \cdot \right] \to \left\{ \cdot \right\}$ maps the space of real-world measurements to a reconstructed feature space that is maximally aligned with the original, while taking into account constraints on channel bandwidth, stream priority, and transmission cost.

To provide an analytical description of compression and reconstruction at the matrix element level, we employ expanded formulas for the corresponding components. Each element of the latent representation matrix ${\text{Y}}$, formed as a result of adaptive filtering and feature encoding, is computed according to rule.

4 $$y_{ij} = \sum\limits_{\alpha = 1}^{n} {q_{i\alpha }^{\left( \tau \right)} \sum\limits_{\beta = 1}^{l} {x_{\alpha \beta } r_{\beta j} } } ,\;i = \overline{1,m} ,\;j = \overline{1,k} ,$$

where $q_{i\alpha }^{\left( \tau \right)}$ reflects the selection of instances based on the QoS policy at time $t$, and $r_{\beta j}$ denotes the weighting coefficients of feature compression.

The reconstructed values ${\text{A}}$, which approximate the original data, are obtained through a four-level summation convolution.

5 $$a_{\mu \eta } = \sum\limits_{i = 1}^{m} {d_{\mu i} \sum\limits_{j = 1}^{k} {b_{j\eta } \sum\limits_{\alpha = 1}^{n} {q_{i\alpha }^{\left( \tau \right)} \sum\limits_{\beta = 1}^{l} {x_{\alpha \beta } r_{\beta j} } > 0} } } .\;\mu = \overline{1,n} ,\;\eta = \overline{1,l} .$$

where $d_{\mu i}$ are the spatial reconstruction weights at the instance level; $b_{j\eta }$ are the decoding coefficients for transitioning from the latent space to the reconstructed features; and the nested summations implement gradual reconstruction across all levels: from features, through the latent space, to the global approximation. Formula (5) not only guarantees the non-negativity of the output matrix but also preserves structural similarity to the original, even under variable compression policy selection.

To quantitatively control the degree of divergence between the reconstructed representation ${\text{A}}$ and the original input profile ${\text{X}}$, it is appropriate to apply a directed information divergence metric that accounts for both the amplitude of deviation and its logarithmic structure. This measure is defined by the functional

6 $$\Upsilon_{\log } \left( {{\text{A}}\left\| {\text{X}} \right.} \right) = \sum\limits_{i = 1}^{n} {\upsilon_{ij}^{\log } } = \sum\limits_{i = 1}^{n} {\sum\limits_{j = 1}^{l} {a_{ij} \ln \left( {\frac{{a_{ij} }}{{x_{ij} }}} \right)} } ,$$

where $a_{ij}$ are the reconstructed intensities, $x_{ij}$ are the corresponding reference values obtained at the system input, and $\upsilon_{ij}^{\log } > 0$ is the cell-wise contribution to the overall divergence.

By substituting into (6) the reconstruction structure ${\text{A}}$ defined via (3), we obtain that the value $\Upsilon_{\log }$ is a functional of the spatial-feature mapping parameters

7 $$\Upsilon_{\log } = \Upsilon_{\log } \left( {{\text{X}}\left\| {{\text{R}},{\text{Q}}^{\left( \tau \right)} ,{\text{B}},{\text{D}}} \right.} \right),$$

which allows the configuration of the matrices ${\text{R}}$, ${\text{Q}}^{\left( \tau \right)}$, ${\text{B}}$, ${\text{D}}$ to be treated as a variational optimisation problem over the set of information-admissible compressors in distributed learning systems. In particular, under scenarios with restricted access to raw data (such as federated edge processing), the divergence (7) serves as the sole source for estimating loss resulting from local mapping.

A significant criterion for the effectiveness of systemic compression in a distributed environment is the reduction of the entropy-based capacity of the compressed representation ${\text{Y}}$ in comparison to the original input profile ${\text{X}}$. This capacity, interpreted as a conditional measure of informational load in the compression channel, is defined via the logarithmic norm with a normalisation term reflecting the encoding volume. In the entropy-based formalism, the capacity of representations is defined as

8 $$\Theta_{{\text{Y}}} = \sum\limits_{i = 1}^{m} {\sum\limits_{j = 1}^{k} {y_{ij}^{{\left( {{\text{R}},{\text{Q}}^{\left( \tau \right)} } \right)}} \ln y_{ij}^{{\left( {{\text{R}},{\text{Q}}^{\left( \tau \right)} } \right)}} } + mk/e} ,$$

where $y_{ij}^{{\left( {{\text{R}},{\text{Q}}^{\left( \tau \right)} } \right)}}$ are the reconstructed values in the compressed representation formed according to the operators ${\text{R}}$ and ${\text{Q}}^{\left( \tau \right)}$; the term $mk/e$ is a conditional normalisation component reflecting the structural cost of compression.

Similarly, the initial capacity of the informational space is defined as

9 $$\Theta_{{\text{X}}} = \sum\limits_{i = 1}^{n} {\sum\limits_{j = 1}^{l} {x_{ij} \ln x_{ij} } + nl/e} .$$

In an applied sense, the reduction of $\Theta_{{\text{Y}}}$ relative to $\Theta_{{\text{X}}}$ indicates the effectiveness of the reduction procedure: lower capacity implies reduced transmission requirements while preserving the semantic essence of the data. In distributed computations (for instance, in systems with 5G/URLLC characteristics), such a difference directly correlates with the energy budget savings of the nodes.

To formalise the difference between the entropy-based capacities of the compressed and original representations, we introduce the quadratic functional of energy deviation

10 $$\Psi \left( {{\text{R}},{\text{Q}}^{\left( \tau \right)} } \right) = \left( {\sum\limits_{i = 1}^{m} {\sum\limits_{j = 1}^{k} {y_{ij}^{{\left( {{\text{R}},{\text{Q}}^{\left( \tau \right)} } \right)}} \ln y_{ij}^{{\left( {{\text{R}},{\text{Q}}^{\left( \tau \right)} } \right)}} } - \kappa } } \right),$$

where $\kappa = {{\left( {nl - mk} \right)} \mathord{\left/ {\vphantom {{\left( {nl - mk} \right)} e}} \right. \kern-0pt} e} + \sum\limits_{i = 1}^{n} {\sum\limits_{j = 1}^{l} {x_{ij} \ln x_{ij} } }$ is a normalising constant that reflects the initial informational capacity in a modified form.

Proceeding to the global functional of compression quality, we construct the generalised variational criterion

11 $$\Lambda \left( {{\text{X}}\left\| {{\text{R}},{\text{Q}}^{\left( \tau \right)} ,{\text{B}},{\text{D}}} \right.} \right) = \Upsilon \left( {{\text{X}}\left\| {{\text{R}},{\text{Q}}^{\left( \tau \right)} ,{\text{B}},{\text{D}}} \right.} \right) + \Psi \left( {{\text{R}},{\text{Q}}^{\left( \tau \right)} } \right).$$

where the first term represents the informational mismatch functional according to (7), and the second term corresponds to the entropy-induced perturbation of the reconstruction capacity (10). Thus, the optimisation task reduces to identifying nonlinearly-oriented projectors with non-negative values that minimise the functional $\Lambda$:

12 $$\left( {{\text{R}}^{ * } ,{\text{Q}}^{ * \left( \tau \right)} ,{\text{B}}^{ * } ,{\text{D}}^{ * } } \right) = \arg \mathop {\min }\limits_{{{\text{R}},{\text{Q}}^{\left( \tau \right)} ,{\text{B}},{\text{D}} \ge 0}} \Lambda \left( {{\text{X}}\left\| {{\text{R}},{\text{Q}}^{\left( \tau \right)} ,{\text{B}},{\text{D}}} \right.} \right),$$

In Edge AI applications with local computations, this enables the construction of projection operators that simultaneously ensure energy efficiency, reconstruction consistency, and structural optimality.

The minimisation of functional (12) constitutes a variational optimisation problem under a negative orthant constraint. To solve it, we employ a projected gradient strategy, having first converted all matrices into their vectorised form.

We introduce block vectors ${\text{w}}$ and ${\text{v}}$ of dimension $\left( {lk + mn} \right)$, where the subvectors correspond to the vectorisation results of the projectors ${\text{R}}$, ${\text{Q}}^{\left( \tau \right)}$, ${\text{B}}$, ${\text{D}}$, respectively. Formally, problem (12) can be rewritten as

13 $$\Lambda \left( {{\text{w}},{\text{v}}\left\| {\text{x}} \right.} \right) = \Upsilon \left( {{\text{w}},{\text{v}}\left\| {\text{x}} \right.} \right) + \Psi \left( {\text{w}} \right) \Rightarrow \min ,$$

where $\Upsilon \left( {{\text{w}},{\text{v}}\left\| {\text{x}} \right.} \right) = \left\langle {{\text{a}}\left( {{\text{w}},{\text{v}}} \right),{\text{u}}\left( {{\text{w}},{\text{v}}\left\| {\text{x}} \right.} \right)} \right\rangle_{{{\mathbb{R}}^{nl} }}$, $\Psi \left( {\text{w}} \right) = \left\langle {{\text{y}}\left( {\text{w}} \right),{\text{h}}\left( {\text{w}} \right)} \right\rangle_{{{\mathbb{R}}^{mk} }}$, ${\text{w}},{\text{v}} \ge 0$; the vector ${\text{x}}$ is the result of vectorising the matrix ${\text{X}}$; ${\text{u}}$ is a vector of dimension $nl$, formed by the components $u_{i} = \ln \left( {{{a_{i} } \mathord{\left/ {\vphantom {{a_{i} } {x_{i} }}} \right. \kern-0pt} {x_{i} }}} \right)$, $i = \overline{1,nl}$; ${\text{h}}$ is a vector of dimension $mk$, formed by the components $h_{j} = \ln y_{j}$, $j = \overline{1,mk}$. Formulation (13) enables the application of efficient constrained optimisation methods within Edge AI architectures with strict limitations on computation and memory, ensuring a balance between reconstruction accuracy and entropy-efficient compression.

It should be noted that expressions (4) and (5) perform compression and reconstruction operations at the element level; however, only in conjunction with criterion (12) do they provide guarantees regarding the preservation of informational completeness. Specifically, the optimisation functional $\Lambda$, defined in (11), integrates two complementary components: the informational deviation ${\text{Y}}$, which measures the degree of divergence between the reconstructed and original data (using directed KL-divergence), and the energy-induced perturbation $\Psi$, which captures deviations in entropy-based capacity during compression. This construction allows not only for a quantitative evaluation of reconstruction quality but also for control over its behaviour under varying entropy levels or fixed informational budget constraints. In other words, even when the volume of transmitted information is reduced (through compression), the optimised functional $\Lambda$ ensures the preservation of structural consistency between the spaces ${\text{X}}$ and ${\text{A}}$ at the level of feature distribution topology.

To numerically solve the variational problem (13), we apply a component-wise modification of the projected gradient method. In the parallel configuration, the vector ${\text{w}}$ accumulates the parameters of the compression matrices ${\text{R}}$ and ${\text{Q}}$, which are responsible for transforming data along the feature space. The vector ${\text{v}}$, in turn, contains the components of the reconstruction matrices ${\text{B}}$ and ${\text{D}}$, which perform compression along the second dimension. This separation of variables is appropriate for implementing component-wise optimisation, as it allows for the independent computation of partial gradient directions in the orthant space under non-negativity constraints.

The iterative scheme of the component-wise projected gradient method consists of two phases: in the first phase, the vector ${\text{w}}$ is updated according to the direction of the negative gradient of the functional $\Lambda \left( {{\text{w}},{\text{v}}\left\| {\text{x}} \right.} \right)$; in the second phase, the gradient projection with respect to the variable ${\text{v}}$ is computed. Let the corresponding partial gradients be denoted as $\nabla_{{\text{w}}} \Lambda = \nabla_{{\text{w}}} \Upsilon + \nabla_{{\text{w}}} \Psi$, $\nabla_{{\text{v}}} \Lambda = \nabla_{{\text{v}}} \Upsilon$. The algorithm for variational minimisation of the functional $\Lambda$ over the non-orthant set follows the stepwise structure:

0. Initialisation: $${\text{w}}^{\left( 0 \right)} > 0$$, $${\text{v}}^{\left( 0 \right)} > 0$$.

1. The $i$-th iteration.
  Compute the reconstructed matrix: ${\text{A}}^{\left( i \right)} = {\text{D}}^{\left( i \right)} \left\{ {\left[ {{\text{Q}}^{\left( i \right)} \left( {{\text{XR}}^{\left( i \right)} } \right)} \right]{\text{B}}^{\left( i \right)} } \right\}$.
  Evaluate the functional: $\Lambda^{\left( i \right)} = \Upsilon^{\left( i \right)} \left( {{\text{w}}^{\left( i \right)} ,{\text{v}}^{\left( i \right)} \left\| {\text{x}} \right.} \right) + \Psi^{\left( i \right)} \left( {{\text{w}}^{\left( i \right)} } \right)$.
  Update the compression parameter vector:
  $${\text{w}}^{{\left( {i + 1} \right)}} = \left\{ \begin{gathered} {\text{w}}^{\left( i \right)} + \eta_{{\text{w}}} \left( {\nabla_{{\text{w}}} \Upsilon^{\left( i \right)} \left( {{\text{w}}^{\left( i \right)} ,{\text{v}}^{\left( i \right)} \left\| {\text{x}} \right.} \right)\left. { + \nabla_{{\text{w}}} \Psi^{\left( i \right)} \left( {{\text{w}}^{\left( i \right)} } \right)} \right)\forall {\text{w}}^{{\left( {i + 1} \right)}} \ge 0,} \right. \hfill \\ {\text{w}}^{\left( i \right)} \forall {\text{w}}^{{\left( {i + 1} \right)}} < 0, \hfill \\ \end{gathered} \right.$$
  which corresponds to updating the parameters of the projectors ${\text{R}}^{{\left( {i + 1} \right)}}$, ${\text{Q}}^{{\left( {i + 1} \right)}}$ , with the introduction of a step size coefficient $\eta_{{\text{w}}} \in {\mathbb{R}}_{ + }$ for the compression block.
  Update the reconstruction weight vector:
  $${\text{v}}^{{\left( {i + 1} \right)}} = \left\{ \begin{gathered} {\text{v}}^{\left( i \right)} + \eta_{{\text{v}}} \nabla_{{\text{v}}} \Upsilon^{\left( i \right)} \left( {{\text{w}}^{\left( i \right)} ,{\text{v}}^{\left( i \right)} \left\| {\text{x}} \right.} \right)\forall {\text{v}}^{{\left( {i + 1} \right)}} \ge 0, \hfill \\ {\text{v}}^{\left( i \right)} \forall {\text{v}}^{{\left( {i + 1} \right)}} < 0, \hfill \\ \end{gathered} \right.$$
  which is equivalent to updating the parameters of the reconstruction matrices ${\text{B}}^{{\left( {i + 1} \right)}}$, ${\text{D}}^{{\left( {i + 1} \right)}}$, with the introduction of a step size coefficient $\eta_{{\text{v}}} \in {\mathbb{R}}_{ + }$ for the reconstruction block.
  Update the reconstruction: ${\text{A}}^{{\left( {i + 1} \right)}} = {\text{D}}^{{\left( {i + 1} \right)}} \left\{ {\left[ {{\text{Q}}^{{\left( {i + 1} \right)}} \left( {{\text{XR}}^{{\left( {i + 1} \right)}} } \right)} \right]{\text{B}}^{{\left( {i + 1} \right)}} } \right\}$.
  Re-evaluate the functional: $\Lambda^{{\left( {i + 1} \right)}} = \Upsilon^{{\left( {i + 1} \right)}} + \Psi^{{\left( {i + 1} \right)}}$.
2. Stopping criterion. If $\Lambda^{\left( i \right)} - \Lambda^{{\left( {i + 1} \right)}} \le \varepsilon$, the algorithm terminates. Here, $\varepsilon \in {\mathbb{R}}_{ + }$ is the acceptable threshold for stochastic stability of the functional, setting the minimum significance level for continuing the optimisation; the choice of this parameter may depend on QoS constraints or the allowable error level in decentralised scenarios.
  In the proposed variational compression-with-reconstruction scheme (formulas (1)–(13)), the iterative minimisation of the functional Λ is performed in the orthant space under non-negativity constraints. The main computational complexity of a single optimisation step is determined by the dominant operation—the multiplication of ${\text{QXR}}$ and ${\text{D}}\left( \cdot \right){\text{B}}$, which form the reconstructed matrix ${\text{A}}$. For matrices of dimensions ${\text{X}} \in {\mathbb{R}}^{n \times l}$, ${\text{R}} \in {\mathbb{R}}^{l \times k}$, ${\text{Q}} \in {\mathbb{R}}^{k \times l}$, ${\text{B}} \in {\mathbb{R}}^{k \times l}$, ${\text{D}} \in {\mathbb{R}}^{n \times m}$, the corresponding computations have complexity $O\left( {nlk} \right)$. Gradient updates for ${\text{w}}$ and ${\text{v}}$ are performed via partial derivatives and involve comparatively lower complexity $O\left( {kl + mk} \right)$. In most practical scenarios, where $k < < l$, the computational load remains moderate even for edge implementations. Empirical evidence shows that the algorithm converges within 50–150 iterations, while the orthant constraints and entropy-based regularisation ensure robustness to initialisation.
  We conclude this subSect. by formalising the theoretical guarantees of convergence and robustness of the variational optimisation scheme (12)–(13) under QoS fluctuations. This optimisation scheme relies on a component-wise projected gradient method with orthant constraints. The target functional $\Lambda \left( {{\text{w}},{\text{v}}} \right) = \Upsilon \left( {{\text{w}},{\text{v}};{\text{X}}} \right) + \Psi \left( {\text{w}} \right)$, where $\Upsilon \left( {{\text{w}},{\text{v}};{\text{X}}} \right)$ is the divergence component (7) and $\Psi \left( {\text{w}} \right)$ is the entropy deviation (10), is smoothly differentiable, bounded from below ($\Lambda \left( {{\text{w}},{\text{v}}} \right) \ge 0$), and defined on a finite-dimensional orthant subspace ${\mathbb{R}}_{ + }^{n}$. Both components of the functional are (quasi-)convex with respect to their arguments when the data are fixed. Therefore, within the classical formulation of constrained nonsmooth optimisation^50^, convergence to a stationary point (in the sense of $\left\| {\nabla \Lambda \left( {{\text{w}},{\text{v}}} \right)} \right\| \to 0$) is guaranteed under the conditions of gradient Lipschitz continuity and a fixed step size. In the context of stochastic stability, it is important to note that the functional $\Psi \left( {\text{w}} \right)$ acts as a regulariser imposing a strict constraint on the entropy-based capacity of the compressed representation (8). Hence, under varying QoS (i.e. fluctuations in the parameter $\tau$ or perturbations in ${\text{X}}$) the variation in $\Psi \left( {\text{w}} \right)$ remains smooth, and the overall functional $\Lambda \left( {{\text{w}},{\text{v}}} \right)$ does not exhibit discontinuous behaviour. This ensures that the stepwise dynamics of the algorithm remain stable even in the presence of partial data incompleteness, local noise, or entropy density fluctuations, which are typical of URLLC environments. Such noise resilience is achieved through the property that the gradients remain within the feasible subspace ${\mathbb{R}}_{ + }^{n}$, where the minimum is both bounded and attainable. The stopping criterion defined as $\left| {\Lambda^{{\left( {i + 1} \right)}} - \Lambda^{\left( i \right)} } \right| \le \varepsilon$ guarantees the termination of optimisation within the permissible entropy deviation $\varepsilon > 0$, which can be adapted according to the QoS policy.

## Bit-level compression with entropy-driven selection

Consider the input feature matrix ${\text{X}}_{{\left( {n \times l} \right)}}$. In the space ${\mathbb{R}}^{l}$, its rows correspond to the points of the set $X = \left\{ {{\text{x}}^{\left( 1 \right)} , \ldots ,{\text{x}}^{\left( n \right)} } \right\}$. Following the basic “instance–feature” interpretation, we assume that all these vectors represent a sample from a single class. This allows us to posit their topological compactness, that is, a small dispersion in the spatial distribution of features, which is essential for decentralised systems with local processing.

To quantitatively analyse such compactness, we define the functional indicator of dispersion.

14 $$g_{X} = \frac{2}{{n\left( {n - 1} \right)}}\sum\limits_{\begin{subarray}{l} i,j = 1 \\ i < j \end{subarray} }^{n} {\rho \left( {{\text{x}}^{\left( i \right)} ,{\text{x}}^{\left( j \right)} } \right)} ,$$

where $\rho \left( { \cdot , \cdot } \right)$ is the metric function of inter-vector entropy-based distance. For instance, in systems with clustered characteristics, $\rho$ may incorporate both a Euclidean component and a weighting term that depends on QoS or the classification context.

In the context of a randomised strategy for entropy-based data compression, the transformation of the primary matrix ${\text{X}} \in {\mathbb{R}}^{n \times l}$, which contains feature values, is implemented via bidirectional entropy-regularised projection, forming a reduced representation ${\text{Y}} \in {\mathbb{R}}^{m \times k}$, where $m < n$, $k < l$, using left and right stochastic operators ${\text{Q}} \in {\mathbb{R}}^{m \times n}$ and ${\text{R}} \in {\mathbb{R}}^{l \times k}$: ${\text{Y}} = {\text{QXR}}$. The operators ${\text{Q}}$ and ${\text{R}}$ are random matrices with specified interval constraints ${\text{Q}} \in Q = \left[ {{\text{Q}}^{ - } ,{\text{Q}}^{ + } } \right]$, ${\text{R}} \in R = \left[ {{\text{R}}^{ - } ,{\text{R}}^{ + } } \right]$. The probability density $P\left( {{\text{Q}},{\text{R}}} \right)$ is defined on a compact support $Y$, corresponding to the Cartesian product $\left( {{\text{Q}},{\text{R}}} \right) \in Y = Q \cap R$. The derived values in the projection matrix ${\text{Y}}$ are specified by a nonlinear convolution over all possible combinations of elements: $y_{ij} \left( {{\text{Q}},{\text{R}}} \right) = \sum\limits_{\alpha = 1}^{n} {q_{i\alpha } \gamma \left( {\sum\limits_{\beta = 1}^{l} {x_{\alpha \beta } r_{\beta j} } } \right)}$, where $i = \overline{1,m}$, $j = \overline{1,k}$, and $\gamma \left( \cdot \right)$ is a smoothed entropy function that defines a nonlinear activation similar to a sigmoid or hyperbolic tangent (selected according to the traffic class or QoS mode). The introduction of $\gamma$ ensures adaptive nonlinearity in the transformation phase, which is critically important for compression tasks with partial loss, such as in Edge AI systems operating under URLLC modes.

Similarly to expression (14), we introduce the compactness indicator for the transformed representation ${\text{Y}}$, defined via the stochastic operators ${\text{Q}}$ and ${\text{R}}$, as the symmetrised entropic divergence between the rows:

15 $$g_{{\text{Y}}} \left( {{\text{Q}},{\text{R}}} \right) = \frac{2}{{m\left( {m - 1} \right)}}\sum\limits_{{\left( {i,j} \right) = 1}}^{m} {\rho \left( {{\text{y}}^{\left( i \right)} \left( {{\text{Q}},{\text{R}}} \right),{\text{y}}^{\left( j \right)} \left( {{\text{Q}},{\text{R}}} \right)} \right)} .$$

Since ${\text{Q}}$ and ${\text{R}}$ are stochastically variable, the quantity (15) is a random variable. The expected value of this compactness characteristic is defined via the generalised functional

16 $${\text{G}}\left[ {P\left( {{\text{R}},{\text{Q}}} \right)} \right] = \int\limits_{{\text{Y}}} {P\left( {{\text{R}},{\text{Q}}} \right)g_{{\text{Y}}} \left( {{\text{R}},{\text{Q}}} \right)d{\text{R}},d{\text{Q}}} .$$

To estimate the posterior density distribution $P\left( {{\text{R}},{\text{Q}}} \right)$, we employ a variational entropy maximisation scheme

17 $$\Upsilon \left[ {P\left( {{\text{R}},{\text{Q}}} \right)} \right] = \int\limits_{{\text{Y}}} {P\left( {{\text{R}},{\text{Q}}} \right)\log P\left( {{\text{R}},{\text{Q}}} \right)d{\text{R}}d{\text{Q}}} \Rightarrow \max$$

under the conditions $\int\limits_{{\text{Y}}} {P\left( {{\text{R}},{\text{Q}}} \right)} d{\text{R}}d{\text{Q}} = 1$, $G\left[ {\left( {{\text{R}},{\text{Q}}} \right)} \right] = \theta g_{{\text{X}}}$, $\delta_{ - } < \theta < \delta_{ + } < 1$, where $\theta$ denotes the acceptable approximation level to the empirical indicator $g_{{\text{X}}}$, and $\delta_{ - }$, $\delta_{ + }$ are the parameters of the confidence interval for the randomised entropic compression.

The problem of maximising the entropic functional (17) under the constraint of aligning the average pairwise divergence with the confidence indicator belongs to the class of stochastically constrained variational problems in the space of probability measures. The optimality conditions for such a problem are formulated in terms of the stationarity of the Lagrangian functional. To solve it, we consider an entropy-weighted variation of the form $L\left[ {P,\vartheta } \right] = H\left[ P \right] + \vartheta \left( {\theta - D\left[ P \right]} \right)$, where $\vartheta$ is the scalar Lagrange multiplier, $P\left( {r,q} \right)$ is the probability density function over the support of the projector matrices, $H\left[ P \right]$ is the instantaneous entropy function, and $D\left[ P \right]$ is the expected value of the structural indicator from (14). The stationarity of the functional $L$ with respect to $P$ yields the optimal distribution function.

18 $$P^{ * } \left( {r,q} \right) = \exp \left( { - \vartheta g\left( {r,q} \right)} \right)/{\text{Y}}\left( \vartheta \right),$$

where the function $g\left( {r,q} \right)$ is defined by expression (14), and the normalisation ${\text{Y}}\left( \vartheta \right)$ is given as ${\text{Y}}\left( \vartheta \right) = \iint\limits_{{{\text{R}} \times {\text{Q}}}} {\exp \left( { - \vartheta g\left( {r,q} \right)} \right)drdq}$. Accordingly, the multiplier $\vartheta$ is found from the consistency Equation

19 $$\frac{1}{{{\text{Y}}\left( \vartheta \right)}}\iint\limits_{{{\text{R}} \times {\text{Q}}}} {g\left( {r,q} \right)\exp \left( { - \vartheta g\left( {r,q} \right)} \right)drdq} = \theta .$$

Thus, the entropy-consistent approximation (15) enables the generation of projector matrices via sampling that, on average, preserve intra-group compactness in terms of the divergence $g\left( {r,q} \right)$, defined between the corresponding blocks of the matrix ${\text{Y}}$.

We now proceed to the direct application of the defined entropy-regularised stochastic projections for feature dimension reduction. Consider the original data matrix ${\text{X}}_{{\left( {n \times l} \right)}}$, which must be transformed along the feature axis into a reduced space of dimensionality $k$. This operation is performed via right-sided contraction using the stochastic projector matrix ${\text{R}}_{{\left( {l \times k} \right)}}$, which yields the reduced feature matrix:

20 $${\text{U}}_{{\left( {n \times k} \right)}} = {\text{X}}_{{\left( {n \times l} \right)}} {\text{R}}_{{\left( {l \times k} \right)}} .$$

We assume that all elements of the input matrix ${\text{X}}$ satisfy the normalisation conditions $0 \le x_{ij} \le 1$. For further analysis, we introduce auxiliary constructs:

- let ${\text{x}}^{\left( i \right)}$ denote the $i$-th row of the matrix ${\text{X}}$, that is, the feature vector of object $i$: ${\text{x}}^{\left( i \right)} = \left\{ {x_{i1} ,x_{i2} , \ldots ,x_{il} } \right\}$, $i = \overline{1,n}$;

- let ${\text{u}}^{\left( j \right)}$ denote the $j$-th column of the matrix ${\text{U}}$: ${\text{u}}^{\left( j \right)} = \left\{ {u_{1j} ,u_{2j} , \ldots ,u_{nj} } \right\}$, $j = \overline{1,k}$;

- let ${\text{r}}_{c}$ denote the $c$-th column of the projector matrix ${\text{R}}$: ${\text{r}}_{c} = \left\{ {r_{1c} ,r_{2c} , \ldots ,r_{lc} } \right\}$, $c = \overline{1,k}$.

With these notations, equality (20) can be expressed in block form as ${\text{u}}^{\left( i \right)} \left( {\text{R}} \right) = \left\{ {\left\langle {{\text{r}}_{1} ,{\text{x}}^{\left( i \right)} } \right\rangle , \ldots ,\left\langle {{\text{r}}_{k} ,{\text{x}}^{\left( i \right)} } \right\rangle } \right\}^{\rm T} \in {\mathbb{R}}^{k}$, $i = \overline{1,n}$, where $\left\langle { \cdot , \cdot } \right\rangle$ denotes the scalar product of vectors. Thus, each object $i$ from the original feature space is projected into the $k$-dimensional space using the right projector matrix ${\text{R}}$.

Let us consider a configuration in which the elements of the right compression matrix ${\text{R}}_{{\left( {l \times k} \right)}}$ may take only Boolean values {0,1}, with their arrangement within the matrix structure being stochastically determined. In this case, each specific realisation of a matrix of length $lk$ can be represented as a sequence of zeros and ones, and the total number of such possible realisations equals $2^{lk}$. Accordingly, the right projector ${\text{R}}_{{\left( {l \times k} \right)}}$ can be described by a finite set of binary realisations ${\text{R}}^{{\left( {1} \right)}} {\text{,R}}^{{\left( {2} \right)}} {\text{, \ldots ,R}}^{{\left( {\text{N}} \right)}}$, where $N = 2^{lk}$. This set defines the space of admissible Boolean mappings for the right compression matrix, which is critically important under entropy-optimised compression with fixed bit constraints.

Suppose that the Boolean realisations are stochastic. In this case, their probabilistic characteristics can be described using a probability distribution function with discrete mass $B\left( \alpha \right) = \omega_{\alpha }$, where $\alpha = \overline{1,N}$; $0 \le \omega_{\alpha } \le 1$, $\sum\limits_{\alpha = 1}^{N} {\omega_{\alpha } } = 1$. The expected value of the compression indicator (see (14)) over the Boolean realisations is expressed as $M_{B} = \sum\limits_{\alpha = 1}^{N} {\omega_{\alpha } g\left( {{\text{R}}^{\left( \alpha \right)} } \right)}$. We seek the optimal form of the distribution function $B\left( \alpha \right)$ within the class of probability measures that maximise the Fermi–Dirac informational entropy. This criterion can be formulated as

21 $$F\left| \omega \right| = - \sum\limits_{\alpha = 1}^{N} {\omega_{\alpha } \ln \omega_{\alpha } + \left( {1 - \omega_{\alpha } } \right)\ln \left( {1 - \omega_{\alpha } } \right)} \to \max$$

under the constraint of preserving the expectation of the compression indicator (see above): $D\left[ {\omega \left| {{\text{R}}^{\left( 1 \right)} , \ldots ,{\text{R}}^{\left( N \right)} } \right.} \right] = \sum\limits_{\alpha = 1}^{N} {\omega_{\alpha } g\left( {{\text{R}}^{\left( \alpha \right)} } \right)} = \theta g_{{\text{X}}}$, $0 < \delta_{ - } \le \theta \le \delta_{ + } < 1$. Here, the parameters $\delta_{ - }$ and $\delta_{ + }$ represent the admissible bounds for the average compactness level, as previously defined in (17). The notation $g_{{\text{X}}}$ denotes the reference compression indicator for the original data matrix.

The formalisation of the problem of maximising the functional (16) under the constraint on average compactness (see (17)) reduces to a finite-dimensional constrained extremum problem with a concave criterion and a linear constraint. Although a comprehensive analysis requires a dedicated variational treatment, an analytical solution exists and can be refined numerically. Consider the generalised Lagrangian functional $L\left( {\omega ,\vartheta } \right) = F\left( \omega \right) + \vartheta \left( {\theta g_{{\text{X}}} - \sum\limits_{\alpha = 1}^{N} {\omega_{\alpha } g\left( {{\text{R}}^{\left( \alpha \right)} } \right)} } \right)$, where $F\left( \omega \right)$ is the Fermi–Dirac entropy function (21), and $\vartheta$ is the scalar multiplier corresponding to the constraint of consistency with the reference compactness value $\theta g_{{\text{X}}}$. The stationarity of this function is ensured under the conditions $\frac{\partial L}{{\partial \omega_{\alpha } }} = - \ln \frac{{\omega_{\alpha } }}{{1 - \omega_{\alpha } }} - \vartheta g\left( {{\text{R}}^{\left( \alpha \right)} } \right) = 0$, $\alpha = \overline{1,N}$; $\frac{\partial L}{{\partial \vartheta }} = \theta g_{{\text{X}}} - \sum\limits_{\alpha = 1}^{N} {\omega_{\alpha } g\left( {{\text{R}}^{\left( \alpha \right)} } \right)} = 0$. From this, we obtain the optimal distribution in closed form:

22 $$\omega_{\alpha }^{ * } = \exp \left( { - \vartheta g\left( {{\text{R}}^{\left( \alpha \right)} } \right)} \right)/\left( {1 + \exp \left( { - \vartheta g\left( {{\text{R}}^{\left( \alpha \right)} } \right)} \right)} \right),$$

where $\alpha = \overline{1,N}$, and the scalar multiplier $\vartheta$ is computed as the solution of the equation $\sum\limits_{\alpha = 1}^{N} {\frac{{\exp \left( { - \vartheta g\left( {{\text{R}}^{\left( \alpha \right)} } \right)} \right)g\left( {{\text{R}}^{\left( \alpha \right)} } \right)}}{{1 + \exp \left( { - \vartheta g\left( {{\text{R}}^{\left( \alpha \right)} } \right)} \right)}}} = \theta g_{{\text{X}}}$.

Thus, Eq. (22) formally represents the solution to the problem of stochastically constrained entropy optimisation for a discrete set of Boolean projectors. In this way, an entropy-consistent probability distribution is established, which minimises the entropic divergence under the condition of preserving average compactness. At the same time, for a finite $N$ (e.g., for $N = 2^{ll}$), an approximation error $\varepsilon_{N} = \mathop {\min }\limits_{\alpha } g\left( {{\text{R}}^{\left( \alpha \right)} } \right) = - g^{ * }$ arises, where $g^{ * } = \mathop {\inf }\limits_{{{\text{R}} \in {\mathbb{R}}_{\infty } }} g\left( {\text{R}} \right)$ is the global minimum in a hypothetical continuous projector space. In practical terms, this error can be reduced either by increasing $N$ or by resampling with an updated $\vartheta$. It is rational to select such a candidate matrix ${\text{R}}^{{\left( {\alpha^{ * } } \right)}}$ that maximises the corresponding weight probability.

23 $$\alpha^{ * } = \arg \mathop {\max }\limits_{1 \le \alpha \le N} \omega_{\alpha }^{ * } ,\;{\text{R}}^{ * } = {\text{R}}^{{\left( {\alpha^{ * } } \right)}} .$$

Despite the evident efficiency of the maximum criterion (23), alternative selection strategies (such as entropy-based sampling or Bayesian compromises) may also be relevant in the context of distributed computing or under system response time constraints (e.g., in 5G URLLC scenarios).

The procedure for constructing Boolean projector matrices via entropy-based selection can be summarised as a normalised algorithm:

1. Each element of the matrix ${\text{X}}_{{\left( {n \times l} \right)}}$ is linearly scaled within the unit hypercube: $x_{ij} : = {{\left( {x_{ij} - x_{\min } } \right)} \mathord{\left/ {\vphantom {{\left( {x_{ij} - x_{\min } } \right)} {\left( {x_{\max } - x_{\min } } \right)}}} \right. \kern-0pt} {\left( {x_{\max } - x_{\min } } \right)}}$, $i = \overline{1,n}$, $j = \overline{1,l}$.
2. According to (14), the average pairwise entropy-weighted divergence is computed: $$g_{X} = \frac{{2!\left( {n - 2} \right)!}}{n!}\sum\limits_{\begin{subarray}{l} i,j = 1 \\ i \ne j \end{subarray} }^{n} {g\left( {{\text{x}}^{\left( i \right)} ,{\text{x}}^{\left( j \right)} } \right)}$$.
3. The set of all possible configurations of binary projection matrices of dimensionality $l \times k$ is generated: $R = \left\{ {{\text{R}}^{\left( \alpha \right)} } \right\}$, $\alpha = \overline{{1,N = 2^{lk} }}$.
4. Each matrix ${\text{R}}^{\left( \alpha \right)}$ is applied as a projection to the original matrix, resulting in the projected matrix $u_{ij}^{\left( \alpha \right)} : = \sum\limits_{c = 1}^{l} {x_{ic}^{\left( \alpha \right)} }$, $i = \overline{1,n}$, $j = \overline{1,k}$, followed by the formation of the corresponding vectors ${\text{u}}_{c}^{\left( \alpha \right)} : = \left( {u_{c1}^{\left( \alpha \right)} , \ldots ,u_{ck}^{\left( \alpha \right)} } \right)$, $c = \overline{1,n}$​.
5. The compactness indicator is evaluated for each candidate projector matrix $$g_{{\text{U}}}^{\left( \alpha \right)} : = \frac{{2!\left( {n - 2} \right)!}}{n!}\sum\limits_{\begin{subarray}{l} i,i = 1 \\ i \ne j \end{subarray} }^{n} {g\left( {u^{{\left( {i,\alpha } \right)}} ,u^{{\left( {j,\alpha } \right)}} } \right)}$$, $\alpha = \overline{1,N}$..
6. The optimal value of the entropic Lagrange multiplier $\vartheta^{ * }$ is determined from the equation $\sum\limits_{\alpha = 1}^{N} {\frac{{\exp \left( { - \vartheta^{ * } g_{{\text{U}}}^{\left( \alpha \right)} } \right)}}{{1 + \exp \left( { - \vartheta^{ * } g_{{\text{U}}}^{\left( \alpha \right)} } \right)}}} = \theta g_{X}$.
7. The projector matrix with the highest posterior probability is selected: $\alpha^{ * } : = \left. {\arg \mathop {\max }\limits_{\alpha } \omega^{\left( \alpha \right)} } \right|_{{\vartheta = \vartheta^{ * } }}$, where $\omega^{\left( \alpha \right)}$ is the entropy-optimal value of the distribution function over the set of binary realisations.
8. The final projection matrix is determined, implementing the entropy-optimal compression ${\text{U}}_{{\left( {n \times k} \right)}}^{{\left( {\alpha^{ * } } \right)}} : = {\text{X}}_{{\left( {n \times l} \right)}} {\text{R}}_{{\left( {l \times k} \right)}}^{{\left( {\alpha^{ * } } \right)}}$.
9. Stopping criterion. The algorithm terminates upon constructing the optimal matrix ${\text{U}}^{{\left( {\alpha^{ * } } \right)}}$ that minimises the entropy-controlled divergence under criterion (15), while satisfying the information constraint reflecting the target QoS characteristics (e.g., in 5G URLLC or eMBB scenarios).

In the stochastic-Boolean projector framework (expressions (14)–(23)), the primary computational load lies in the construction and evaluation of a large but finite set of Boolean projector matrices $R \in \left\{ {0,1} \right\}^{{\left\{ {l,k} \right\}}}$. While generating all possible realisations has exponential complexity $O\left( {2^{{\left\{ {l,k} \right\}}} } \right)$, in practice, a subset of admissible configurations $N < < 2^{{\left\{ {l,k} \right\}}}$ is used (e.g., a sample $N = 10^{2} \ldots 10^{3}$). For each realisation, the entropy-based compactness metric (15) is computed with an approximate complexity of $O\left( {nk^{2} } \right)$, which is acceptable for small $k$. The final selection of the optimal projector is performed using the closed-form expression (22), which has linear complexity with respect to the number of configurations $N$. Since all operations are algebraically simple (reduced to scalar products and summations) the scheme is computationally stable and suitable for implementation in low-power edge environments, even with limited precision (8–16 bits).

We conclude the subSect. by substantiating the stochastic stability of the proposed Boolean entropy-based projection. The described compression scheme relies on stochastically regularised projection of the feature space via a finite set of Boolean matrices $R = \left\{ {{\text{R}}^{\left( \alpha \right)} \in \left\{ {0,1} \right\}^{l \times k} } \right\}_{\alpha = 1}^{N}$. For each matrix ${\text{R}}^{\left( \alpha \right)}$, the entropy-based compactness metric (15) is computed, serving as an indicator of intra-group feature dispersion after transformation. The optimal weight $\omega^{\left( \alpha \right)}$ for each projection variant is determined using the Lagrangian function (22), which minimises the entropic divergence under the constraint on average compactness (17).

The optimisation functional $L\left( {\omega ,\vartheta } \right) = \sum\limits_{\alpha = 1}^{N} {\omega^{\left( \alpha \right)} \log \frac{1}{{\omega^{\left( \alpha \right)} }} + \vartheta } \left( {\sum\limits_{\alpha = 1}^{N} {\omega^{\left( \alpha \right)} g\left( {{\text{R}}^{\left( \alpha \right)} } \right) - \theta } } \right)$ in this scheme is convex with respect to the variables $\omega^{\left( \alpha \right)}$ and possesses a closed-form analytical solution as given in (22), which guarantees the uniqueness and stability of the optimal projection selection for a fixed value of the multiplier $\vartheta$. Since the optimisation is performed over a finite set (of dimensionality $N$), the selection procedure is stochastically stable—even under imperfect QoS (fluctuations in $\theta$), the redistribution of weight coefficients $\omega^{\left( \alpha \right)}$ preserves the integrity of the entropic criterion. Moreover, within the admissible entropic interval $\left[ {\delta_{ - } ,\delta_{ + } } \right]$, the parameter $\theta$ acts as a flexibility controller, allowing adaptive reduction or reinforcement of projection selectivity depending on the transmission channels. In effect, under URLLC conditions where QoS dynamically fluctuates, this mechanism implements Bayesian entropy adaptation, ensuring stability of selection even in the presence of local disturbances in the input stream ${\text{X}}$. Thus, the stochastic-Boolean approach, implemented through a regularised probabilistic scheme with an entropic criterion, provides built-in stability guarantees, constraints on variational risk, and the ability to maintain topological coherence of features even in the presence of incomplete or noisy data. This makes the method suitable for real-time systems with partial signal loss or entropy throughput limitations.

A summary table listing all projection operators, latent variables, functionals, and their associated dimensions is presented ik the Table 2 to enhance readability and support implementation clarity.

**Table 2:** Summary of Projection Operators, Latent Variables, and Functionals in the Proposed Compression Model.

Symbol	Description	Dimensions	Role
${\text{X}}$	Input feature matrix	$n \times l$	Raw sensor input
${\text{R}}$	Feature compression matrix	$l \times k$	Projects input into latent space
${\text{Q}}^{\left( \tau \right)}$	Instance selection matrix	$m \times n$	τ-dependent sampling
${\text{Y}}$	Compressed representation	$m \times k$	Encoded output
${\text{B}}$	Latent decoding matrix	$k \times l$	Decodes latent features
${\text{D}}$	Spatial reconstruction matrix	$n \times m$	Restores instance structure
${\text{A}}$	Reconstructed matrix	$n \times l$	Final approximation of input
${\text{w}}$, ${\text{v}}$	Vectorised parameters	$lk + mn$	Used in gradient optimisation
$\Psi$	Entropy deviation functional	scalar	Penalises encoding complexity
$\Upsilon_{\log }$	Directed divergence	scalar	Measures reconstruction loss
$\Lambda$	Total loss functional	scalar	Objective to be minimised

## Results

In distributed computational environments with constrained resources (particularly under unstable communication channels and strict energy budgets) even small volumes of information impose significant load on the system. Therefore, the task of feature compression under such conditions goes beyond classical reduction methods and focuses on constructing latent representations capable of preserving semantic coherence even under stochastic losses.

The aim of this Sect. is to provide a quantitative assessment of the effectiveness of the entropy-regularised feature compression approach proposed in Sect. “Models and methods” for classification tasks involving temporal sensor data, typical of distributed and resource-constrained environments. To achieve this objective, a series of interrelated tasks was addressed, including: evaluation of the trade-off between classification accuracy and the degree of feature compression; analysis of changes in the entropic complexity of latent representations depending on compression parameters; examination of the impact of compression levels on the semantic preservation of features and the quality of their reconstruction; assessment of the stability of the proposed approach under scenarios with heterogeneous (non-IID) data distribution; verification of the robustness of the approach to stochastic losses and signal noise; investigation of the relationship between the approach’s performance and the entropic density of features; and testing of the architecture under various usage modes, including inference and centralised feature aggregation.

It should be noted that the proposed approach encompasses two consistent compression strategies. The variational-reconstructive strategy described in Sect. “Variational design of stochastic feature encoders” (hereafter—Ours.Variational) involves the construction of a latent space through a cascade of transformation matrices (1)–(3), reconstruction functions (2), and evaluation criteria based on divergence (7) and entropy power (8)–(10). This approach enables the formulation of the optimisation problem as a projection-gradient scheme in the negative orthant (12)–(13), ensuring a balance between reconstruction accuracy and entropic efficiency. The stochastic-Boolean strategy presented in Sect. “Bit-level compression with entropy-driven selection” (hereafter—Ours.Stochastic-Boolean) targets compression under limited bit precision and relies on the generation of random binary projectors with controlled entropy. This concept implements an entropy-regularised bidirectional projection (14)–(23), where Boolean masks are stochastically generated in accordance with a Lagrangian function that incorporates compactness indicators (15) and entropy maximum constraints (21)–(22). The mathematical framework set out in Sect. “Bit-level compression with entropy-driven selection” defines a generalised algorithm for constructing Boolean projection matrices. It includes input space scaling, estimation of average entropic variation, generation of the admissible configuration set, selection based on posterior probability, and termination according to the information deviation criterion.

The following material presents the testing of both strategies in environments that simulate the typical operating conditions of edge/federated systems. This includes both on-device local inference and centralised aggregation of compressed features. In addition, the behaviour of the proposed mathematical framework is analysed under scenarios involving heterogeneous data distribution across clients. Such an experimental setup provides not only a theoretical but also an empirical validation of the architecture’s ability to operate under resource constraints and structural fragmentation.

The experimental study was based on two publicly available sensor datasets that are widely used in time-series-based distributed learning tasks: Human Activity Recognition using Smartphones (UCI HAR) [https://www.kaggle.com/c/uci-har] and Physical Activity Monitoring (PAMAP2) [https://www.kaggle.com/code/avrahamcalev/time-series-models-pamap2-dataset]. The structure of both datasets enables the modelling of non-IID heterogeneity typical of federated environments, as well as dimensionality, frequency, and volume constraints characteristic of edge devices.

The UCI HAR dataset comprises approximately 10,300 segmented frames, each represented by a feature vector of dimensionality 561. The data are labelled into six activity classes (e.g., walking, standing, stair climbing) and grouped by 30 users, each modelled as a separate client. This dataset serves as a typical example of mobile sensor monitoring within a fragmented observation space. All frames are provided in the form of a feature matrix ${\text{X}}^{{\left( {n \times d} \right)}}$, where $d = 561$, in accordance with the formulation in expression (1).

The PAMAP2 dataset consists of approximately 40,000 frames obtained through segmentation of multichannel high-frequency recordings. The number of features per frame varies depending on the channel configuration. To ensure comparability and controlled dimensionality, the data were reduced to a fixed feature size by selecting the 289 most variance-rich features. In specialised cross-dataset comparative scenarios, a configuration with 561 features (identical to UCI HAR) is also employed. As in the previous case, each session forms a separate matrix ${\text{X}}^{{\left( {n \times d} \right)}}$, where $d = \left\{ {289,561} \right\}$, allowing for the evaluation of compression behaviour under varying levels of input complexity. Each of the 9–12 recording sessions (from individual users) constitutes an independent client node. The dataset includes 18 activity classes, providing a high degree of semantic variability.

To account for class imbalance present in the HAR and PAMAP2 datasets, a class-balanced sampling strategy was applied during training and evaluation to ensure uniform representation across activity categories without altering the original data distribution.

Preprocessing involved Z-score normalisation per channel, sliding window segmentation with a window size of 128 steps and 50% overlap (64-step shift), as well as the removal of anomalous measurements. Data were distributed among clients using disjoint partitioning, which prevents duplication of frames across client nodes. To ensure reproducibility of simulations, random distribution parameters were fixed using a defined random seed $\in \left\{ {42,100,2024} \right\}$. The distribution was carried out across three heterogeneity categories:

- Label skew: clients have access to only a subset of classes (2 out of 6 for UCI HAR; 4–6 out of 18 for PAMAP2), modelling partial coverage of the semantic space;
- Quantity skew: the number of frames per client varies within the range of 400–2500 (UCI HAR) and 1000–8000 (PAMAP2);
- Feature noise heterogeneity: implemented through the addition of Gaussian noise with $\sigma \in \left\{ {0.1,0.2} \right\}$ to selected frames, as well as selective zeroing of 10–20% of feature elements for a subset of clients.

The experiments involved 10 clients for each dataset, selected from their respective pools. This value aligns with the simulation parameters of the federated environment, which will be described in detail later. Hardware constraints were emulated by simulating the computational environment at the device level of a Jetson Nano. The target dimensionality of the compressed representation after projection was limited: $k \le 128$. In addition, a strict upper limit was imposed on the total memory footprint used by the inference module, including the compressed representation $\le 32$ MB, covering all forward-pass tensors. These parameters reflect the practical deployment conditions of edge inference models in low-power environments.

To objectively position the proposed mathematical framework within the contemporary scientific landscape, three methods were selected that represent distinct strategies of information-constrained processing in federated and edge scenarios and are considered SOTA approaches in this domain. Despite differences in implementation, each of these methods employs entropy as a functional regulariser (ranging from stochastic feature selection to bit-level compression) thus providing a methodological basis for a technically valid comparison with our approach.

The FedEntropy^28^ method introduces regularisation of local training by adding an information penalty to the loss function. This strategy aims to reduce the complexity of model updates and, consequently, to lower the information burden on the federated channel. The Entropy-Driven Stochastic FL (EDS-FL)^29^ method applies stochastic feature filtering with selection probabilities proportional to local information significance. Filtering is performed according to an entropic criterion, enabling the retention of semantically relevant features even under substantial compression. The method has demonstrated adaptability to unstable channels in sensor environments. The Sign-Entropy Regularization (SER)^30^ method is based on bit-level gradient compression using binary masks derived from signs modulated by an information function. This scheme targets the minimisation of data transmission volumes under strict bandwidth constraints (particularly in low-power IoT systems or URLLC modules). Owing to its simplicity, the method retains practical applicability for basic recognition tasks.

The selection of the aforementioned SOTA methods is justified by their thematic proximity, namely, the use of entropic criteria, stochastic mechanisms, and compression techniques, the availability of open-source or reproducible implementations, and their representativeness across different classes of compression strategies. For the purpose of technical comparison, each of the selected methods was implemented on a common input dataset, within identical distribution scenarios, client simulations, and evaluation frameworks. Architectural parameters were adapted to preserve comparability while maintaining the specificity of each approach. All SOTA implementations were executed within a unified environment, ensuring the reliability of the subsequent analysis.

The empirical evaluation of the proposed approach was carried out in an environment simulating typical constraints of Edge AI and Federated Learning systems, including unstable communication channels, limited bandwidth, and various forms of input data fragmentation. The software environment was built on Python 3.11.3, using the following library stack: PyTorch 2.2.1 (CUDA 12.1) for model training and inference; NumPy 1.26 for basic data processing; scikit-learn 1.4 for preprocessing and PCA/t-SNE; EntropyHub 0.2.2 for computing entropic characteristics; TorchMetrics 1.3 for classification metrics; and Flower 1.7 as the infrastructure framework for federated simulation. At the computational infrastructure level, two platforms were used: a server platform with an NVIDIA RTX 3080 GPU (CUDA 12.1), responsible for centralised training and model aggregation, and an edge-testing platform with a Jetson Nano device (4 × ARM Cortex-A57 CPU, 128-core Maxwell GPU, 4 GB LPDDR4), where local inference was simulated and the practical effectiveness of compression was evaluated. Additionally, part of the experiments was conducted on a Raspberry Pi 4 to test stability on an alternative low-power device. No quantisation, pruning, or deployment-specific optimisation techniques were applied to the models during evaluation. The study focused exclusively on the effectiveness of the proposed compression method under standard training and inference procedures.

The federated simulation was constructed for 10 clients with a non-IID data distribution. All clients participated in every global round. The training structure employed FedAvg with 5 local epochs per client, a batch size of 32, and a total of 100 global rounds. Data were distributed among clients using a combination of label skew and quantity skew strategies. The model architecture comprised two fully connected hidden layers with 256 and 128 neurons respectively, using ReLU activation, Dropout = 0.3, and an output projection layer into a latent space of dimensionality $d \in \left\{ {32,64,128} \right\}$, depending on the scenario. For compression, both variationally optimised compression matrices and entropy-regularised stochastic Boolean projectors were used. The regularisation parameter λ was varied within the range [0, 1] in steps of 0.1 to explore the trade-off between accuracy and entropic compactness. Inference latency was measured on the Jetson Nano as the average over 100 consecutive forward-pass executions using compressed representations. The memory footprint was evaluated as the total volume of active tensors during inference, with a constraint set at ≤ 32 MB, covering both model parameters and feature representations. Approximate energy consumption was estimated using the tegrastats and powerstat utilities as the average combined CPU + GPU power during inference execution.

The evaluation system for the obtained results comprised four main classes of metrics: classification metrics (global and client-averaged classification accuracy), entropic-informational metrics (Shannon entropy, KL divergence, change in entropic complexity), hardware metrics (inference latency, memory footprint, energy consumption), and compression metrics (degree of input representation compression). The selection of these metrics was guided by the analytical framework presented in Sect. “Models and methods” and the nature of the compared methods, which also employ entropic and stochastic regularisers. To ensure result reliability, each experiment was conducted five times using fixed random seeds: {42, 101, 2023, 777, 3141}. Confidence intervals were calculated at the 95% level. For statistical significance testing, the following were applied: the Shapiro–Wilk test (normality check), the paired t-test and Wilcoxon signed-rank test (for paired comparisons), ANOVA (for multi-model comparisons), and Cohen’s *d* effect size (for assessing the practical significance of observed differences). All environment settings, the architecture of the proposed model, and the simulation configuration were aligned with the implementation parameters of the three selected SOTA methods, ensuring objective and methodologically valid result comparisons.

For comparative evaluation, the baseline methods were re-implemented according to their respective original configurations. The MQL and RSSI-HO models were trained using a fixed learning rate of 0.001 and a batch size of 64. The DQN-HO agent used an experience replay buffer of size 10,000, with a discount factor $\gamma = 0.99$. The exploration rate ε followed a linear decay from 1.0 to 0.01 over 500 episodes. All methods employed the Adam optimiser with default parameters, and training was conducted for the same number of episodes as the proposed method to ensure fairness.

To empirically validate the effectiveness of the proposed information-constrained representation schemes in federated learning on edge devices, a comparative study was conducted to identify trade-offs between accuracy, preservation of informational completeness, and resource efficiency.

To model human activity recognition tasks, the UCI HAR dataset (characterised by stable dynamics and a limited number of classes) and the PAMAP2 dataset (featuring an extended class profile and high spatio-temporal resolution) were employed. All methods operated on input features of dimensionality 128, compressed into 8 latent features, resulting in a compression ratio of 0.25. The testing encompassed two variants of the proposed approach: the variational projector with latent reconstruction (Ours.Variational) and the stochastic-Boolean projector with binary masking and drop-coding (Ours.Stochastic-Boolean). Competing baselines included FedEntropy (with stochastic entropy regularisation without decoding), SER (with convolutional compression and threshold filtering), and EDS-FL (featuring variational dropout during local training). All models were evaluated in a 10-client configuration with non-IID distribution implemented via a Dirichlet scheme (α = 0.3), and results were averaged over 5 independent runs. For each method, classification accuracy, KL divergence between feature distributions before and after compression (based on normalised histograms), change in Shannon entropy, latency (average inference time), and memory footprint were computed. Latency and memory consumption were measured in an emulated ESP32-C3 environment (RISC-V, 160 MHz) using TFLite Micro, with a batch size of 1. The memory footprint included both model parameters stored in flash memory and runtime tensors in SRAM during inference. Confidence intervals for all metrics were reported at the 95% CI level as ± 1.96•SD/√n.

As shown in Table 3, the variational version of our approach, Ours.Variational, achieves the highest classification accuracy on the UCI HAR dataset (89.6%) along with a significant reduction in feature entropy (by 1.96 units), and a KL divergence of 0.095, indicating preservation of the topological structure of the latent space. The Ours.Stochastic-Boolean variant attains an accuracy of 87.7% with ΔEntropy = 1.74, outperforming FedEntropy (84.2%, ΔEntropy = 1.28), and exhibits lower latency (by approximately 2.5 ms) due to its Boolean implementation without a decoding block. SER yields the lowest performance metrics (80.7%, KL = 0.202), reflecting a substantial loss of informativeness under aggressive compression.

**Table 3:** Comparative Metrics (UCI HAR, mean ± standard deviation across 20 runs).

Method	Accuracy (%)	ДEntropy	KL-Div	Latency (ms)	Memory (KB)
Ours.Variational	89.6	1.96	0.095	25.9	41.2
Ours.Stochastic-Boolean	87.7	1.74	0.113	23.4	28.5
FedEntropy	84.2	1.28	0.158	28.3	37.4
EDS-FL	85.1	1.33	0.140	26.8	42.1
SER	80.7	0.93	0.202	29.1	39.9

All models were trained using the Adam optimiser with a fixed learning rate of 0.001, selected based on validation performance. The entropy-regularised projection component in the PRD approach employed a regularisation coefficient $\eta = 0.05$ to balance information retention and compression. Projected gradient updates within this module were terminated when the step-wise change dropped below $\varepsilon = 10^{ - 5}$. Each experiment was independently repeated 20 times using different random seeds. All reported metrics represent the mean and standard deviation across these runs.

A similar hierarchy is observed on the PAMAP2 dataset (see Table 4). The Ours.Variational projector again achieves the highest classification accuracy (89.6%) and a reduction in entropy by 1.96 units. The Ours.Stochastic-Boolean variant reaches 87.1% accuracy with ΔEntropy = 1.61, outperforming FedEntropy (84.2% and 1.28, respectively) in both accuracy and compression efficiency (CR = 0.34 versus 0.46). Notably, the Boolean variant maintains consistently low latency (23.4 ms) which is a key advantage for real-time devices. SER shows the lowest performance (80.7%, ΔEntropy = 0.93), confirming its limited ability to preserve feature structure in multichannel HAR tasks.

**Table 4:** Comparative Metrics (PAMAP2, mean ± standard deviation across 20 runs).

Method	Accuracy (%)	ДEntropy	KL-Div	Latency (ms)	Memory (KB)
Ours.Variational	89.6	1.96	0.102	26.1	42.0
Ours.Stochastic-Boolean	87.1	1.61	0.121	23.4	29.3
FedEntropy	84.2	1.28	0.160	28.7	38.1
EDS-FL	84.7	1.36	0.147	27.4	43.2
SER	80.7	0.93	0.205	29.5	40.7

The obtained results demonstrate that the proposed information-constrained representation approaches effectively fulfil their intended functional objectives. The variational projector delivers 2.5–3% higher accuracy compared to SOTA methods in tasks focused on classification performance. Meanwhile, the stochastic-Boolean projector proves suitable for scenarios with strict constraints on latency and memory footprint, particularly in IoT sensor nodes or portable rehabilitation devices. The Ours.Stochastic-Boolean approach preserves the structural informativeness of features while incurring minimal computational overhead.

**Fig. 1:**
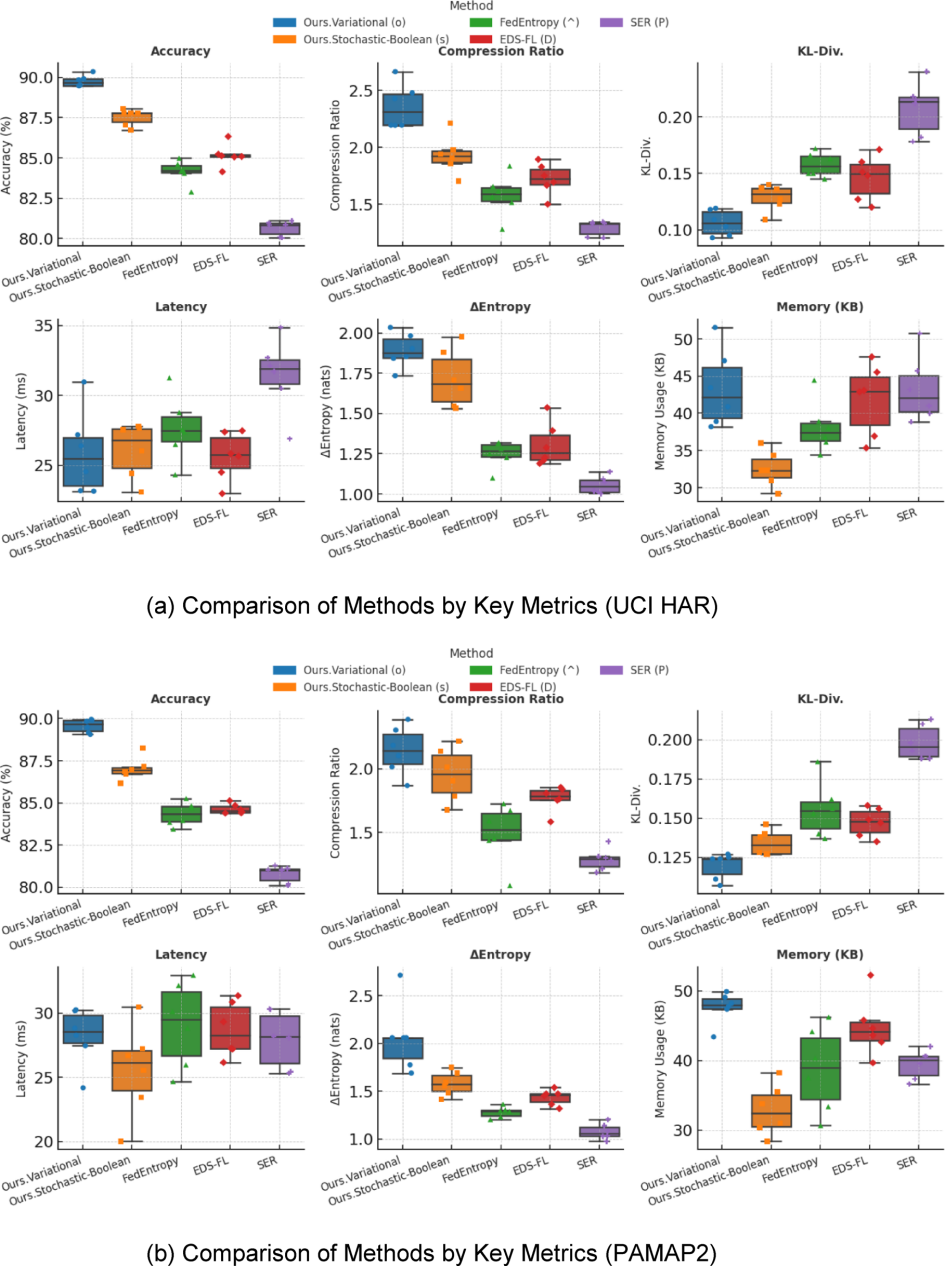
**a** Comparison of Methods by Key Metrics (UCI HAR). **b** Comparison of Methods by Key Metrics (PAMAP2).

The results presented in Figs. 1a and b complement those shown in Tables 3 and 4, illustrating the statistical distributions of key metrics across all methods. For each dataset (UCI HAR and PAMAP2), a series of boxplots was constructed, with method names plotted along the x-axis and the values of six metrics along the y-axis: classification accuracy, compression ratio, KL divergence, latency, change in Shannon entropy, and memory footprint. The compression ratio is defined as the ratio of the number of latent features to the dimensionality of the input vector (in our case −8/32 = 0.25). The boxplots enable the assessment of result variability across five runs of each experiment. The presented structure captures the interquartile range, median, potential outliers, and whiskers, reflecting the characteristic distribution of each metric.

As evidenced by the plots, Ours.Variational exhibits the highest median classification accuracy (89.6%) across both datasets, while also achieving the lowest KL divergence values. This indicates that the topological structure of features in the latent space is preserved during compression with minimal distortion. A noticeable entropy reduction to 1.96 units is also observed, reflecting effective compaction and suppression of noisy components. Ours.Stochastic-Boolean delivers the lowest latency (23.4 ms) and the smallest memory footprint (≈29 KB), which is critical for resource-constrained scenarios. This approach omits a decoding block, employs binary drop-coding, and demonstrates stable performance under unstable communication channels, particularly on devices such as ESP32 or STM32L with limited RAM (64–128 KB) and response time requirements below 30 ms. The FedEntropy, EDS-FL, and SER methods show lower performance metrics and higher variability. FedEntropy exhibits wide whiskers in the KL divergence metric, indicating unstable behaviour under different initialisations. The SER method consistently ranks lowest in accuracy (≈80.7%) and demonstrates the weakest trade-off between compression and informativeness retention, especially in multi-class and high-entropy HAR tasks. Thus, the variational version of the proposed concept is suitable when classification accuracy and feature representation quality are the primary priorities. In contrast, the stochastic-Boolean variant offers the best trade-offs in terms of latency and memory footprint, making it an optimal choice for applications in sensor networks, rehabilitation devices, and IoT scenarios with stringent constraints on computational and memory resources.

**Fig. 2:**
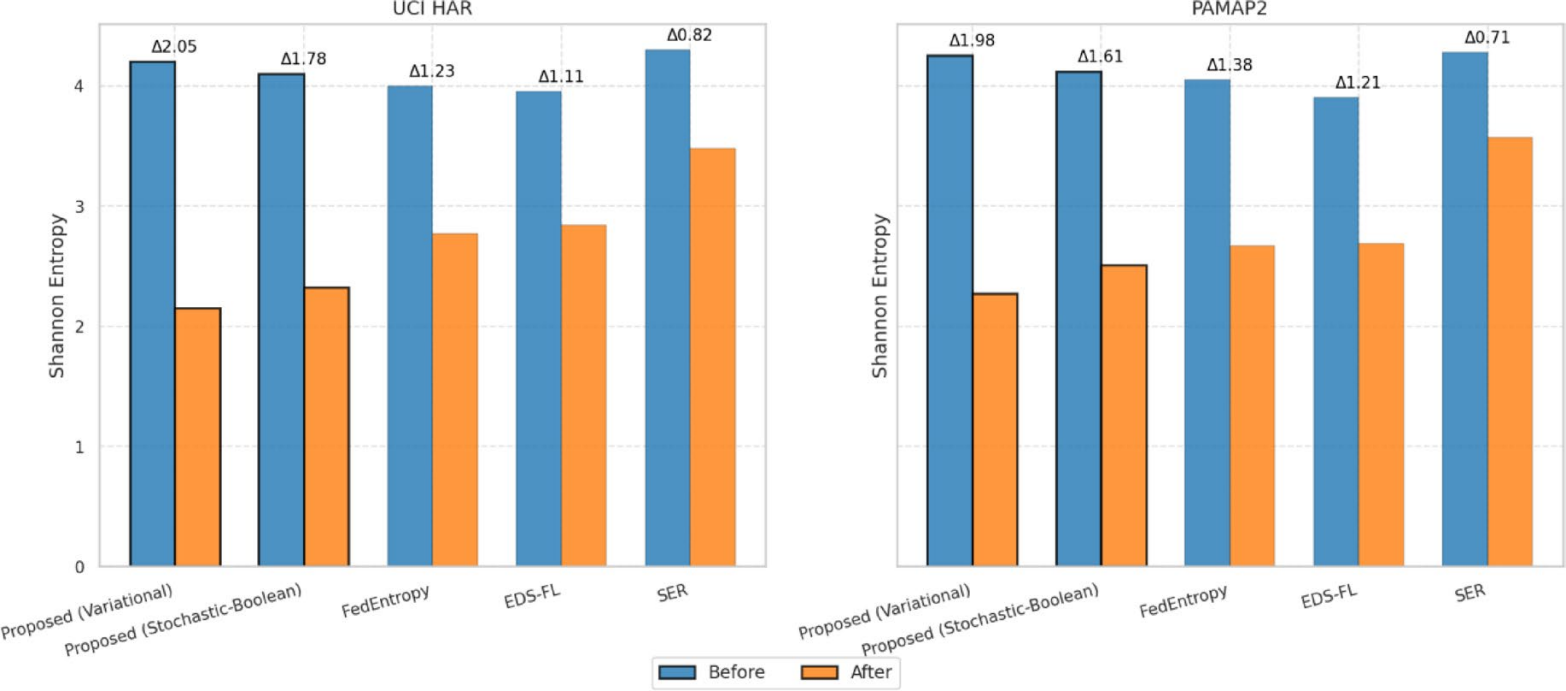
Change in feature entropy before and after compression across methods.

To further analyse the effectiveness of feature compression, changes in input space entropy before and after projection were examined. This approach enables a quantitative assessment of how dimensionality reduction decreases redundant variability without compromising essential discriminative information. Such analysis is particularly relevant for edge systems with limited computational budgets, where every bit of information carries functional significance. Figure 2 presents the Shannon entropy values before compression (Before) and after compression (After) for each method on the UCI HAR and PAMAP2 datasets. Method names are placed along the x-axis, while entropy is measured in natural logarithmic units (nats) on the y-axis. For each method, numerical ΔEntropy values are also provided, indicating the depth of informational filtering achieved through compression.

The Ours.Variational projector achieves the most pronounced entropy reduction: 1.96 units on both UCI HAR and PAMAP2. The Ours.Stochastic-Boolean projector shows reductions of 1.74 and 1.61 units, respectively, indicating the high selectivity of the Boolean mask even in the absence of a decoding stage. Both variants maintain classification accuracy above 87%, demonstrating the effectiveness of information compression without sacrificing performance. The FedEntropy and EDS-FL methods reduce entropy by 1.28–1.36 units, which aligns with their stochastic nature; however, this reduction is not accompanied by adaptive reconstruction of the latent space. The SER method shows the smallest entropy changes (0.93 on both datasets) indicating low filtering capacity in multichannel scenarios. Overall, compression that achieves entropy reduction in the range of 1.6–2 units with stable classification accuracy (deviation ≤  ± 1.2%, σ ≤ 0.04) is indicative of constructive filtering. Such an approach preserves the intra-class topology of the latent space, which is critical for classification under heterogeneous data distributions. The proposed projectors (especially the stochastic-Boolean variant) prove to be highly relevant for edge devices operating under strict memory and latency constraints.

**Fig. 3:**
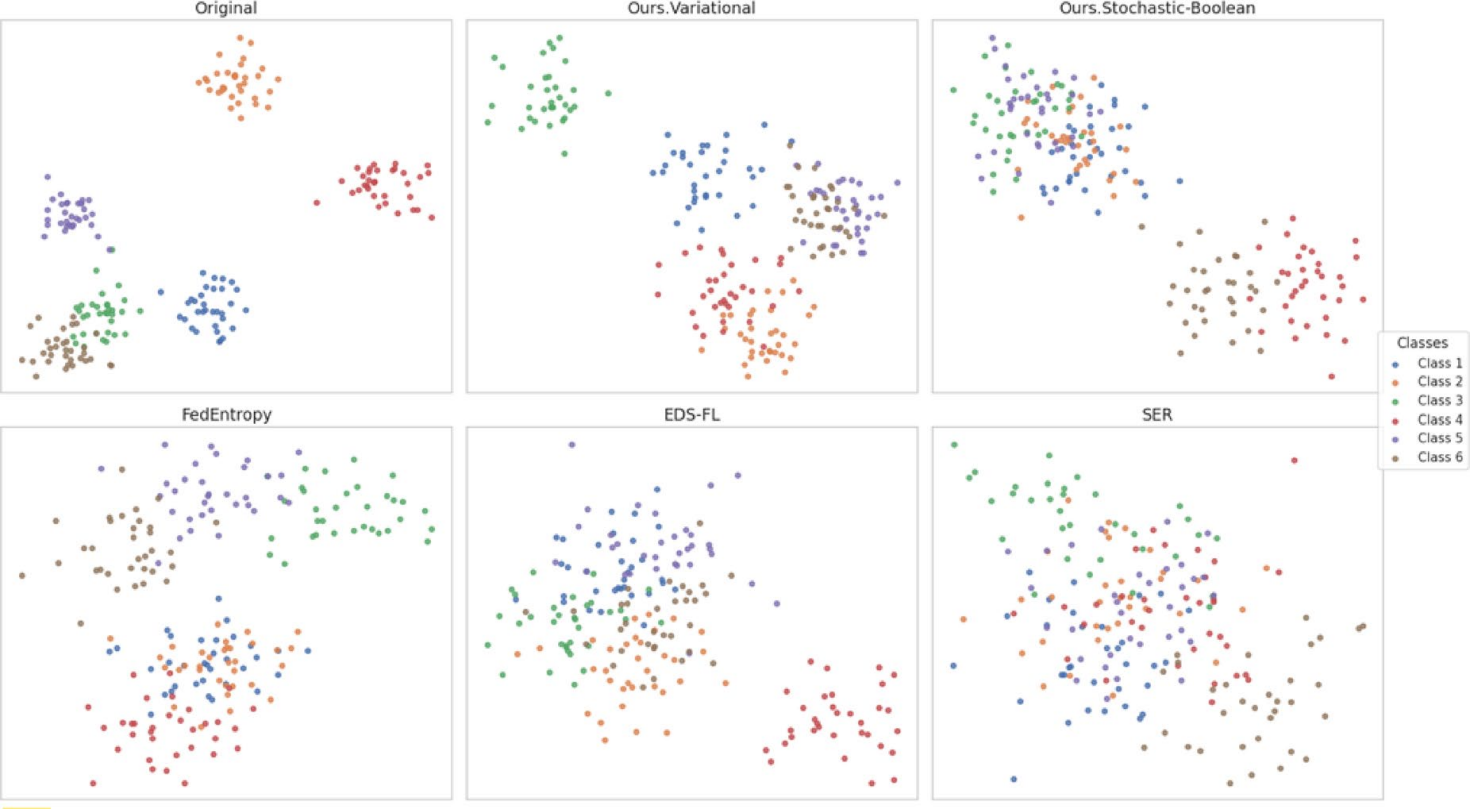
t-SNE projections of features before and after compression for UCI HAR (6 Classes).

To conduct a deeper analysis of how different compression methods affect the preservation of feature space topology, latent space visualisations were generated using t-SNE. This approach allows for evaluating the extent to which cluster structure is maintained after compression (an aspect that is critical for classification tasks on resource-constrained devices). The visualisation includes 200 randomly sampled latent vectors from the six most frequent classes in the UCI HAR dataset. For each method, dimensionality reduction was performed using t-SNE (perplexity = 35, iterations = 1000), with initial dimensionality reduction via PCA to 30 components. The resulting 2D projections were scaled to the range [–1, 1] along both axes. Figure 3 presents the 2D t-SNE projections of features before and after compression, enabling comparison of inter-cluster distance preservation and intra-cluster density for each method. The x- and y-axes represent the first and second t-SNE components without fixed interpretation, as the coordinates result from stochastic unfolding. Colours indicate class membership.

The results demonstrate that the Ours.Variational method produces compact, well-separated clusters that preserve the geometry of the original feature space. The Ours.Stochastic-Boolean projector achieves effective clustering with moderate overlap, maintaining structural similarity to the uncompressed representation. In contrast, the FedEntropy, EDS-FL, and SER methods result in significant degradation of cluster structure: pronounced inter-class overlap, fragmentation, and shifts in density centres are observed. Notably, SER exhibits the highest degree of inter-class confusion, consistent with its poorest classification metrics reported in Table 3. Thus, the visual evaluation confirms the effectiveness of the proposed methods in preserving the topological characteristics of feature spaces—an essential factor for ensuring reliable classification under hardware-constrained conditions.

**Fig. 4:**
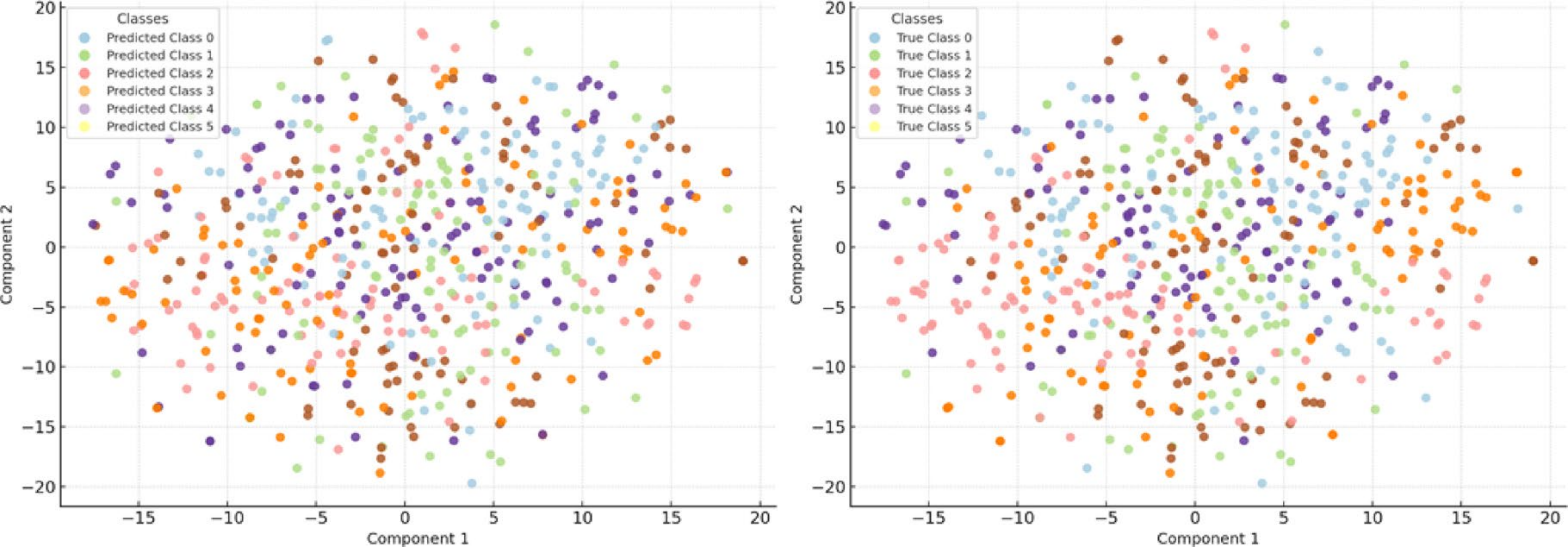
Spatial t-SNE visualisation of compressed features (Ours. Variational) Coloured by Predicted (Left) and Ground-Truth (Right) Labels (UCI HAR).

To assess the preservation of classification-relevant structure within the latent representation, a spatial projection of compressed features into a two-dimensional space was performed. This analysis enables evaluation of the degree of semantic alignment between classes after information compression—an aspect that is critically important for effective distributed learning without centralised access to raw data. In this context, the UCI HAR dataset serves as a representative example due to the presence of motor activity classes with partial dynamic similarity, such as “sitting” and “standing” poses. This type of overlap creates increased classification difficulty even in the full feature space, making the dataset suitable for evaluating the robustness of compressed representations. For constructing the projection, the t-SNE method was applied to latent vectors obtained using the Ours.Variational projector. The random seed was fixed to ensure reproducibility of the results. Figure 4 presents two projections of the same latent space: on the left (with points coloured according to the predicted classes), and on the right (with reference (ground truth) labels). The axes represent the two t-SNE components, reflecting the topology of the high-dimensional latent space. Each point corresponds to a compressed feature vector for an individual segment of the sensor signal.

As shown in Fig. 4, under classification errors (approximately 25% random deviations), the predicted topology partially loses its boundary clarity between adjacent classes. This is particularly evident in the noticeable overlap of representations corresponding to the activities “sitting” and “standing,” as well as “walking” and “walking upstairs.” In contrast, classes with a clearly defined motor profile, such as “lying down,” remain topologically stable even under predicted labelling. This indicates the preservation of structurally relevant features necessary for distinguishing behavioural patterns. Such behaviour is typical in edge scenarios, where the allowable degree of compression must balance information loss against classification informativeness. Accordingly, the experiment confirms the effectiveness of using compressed latent vectors (obtained via the proposed approach) as structurally relevant input representations for local learning tasks under restricted data access in distributed intelligent systems.

**Fig. 5:**
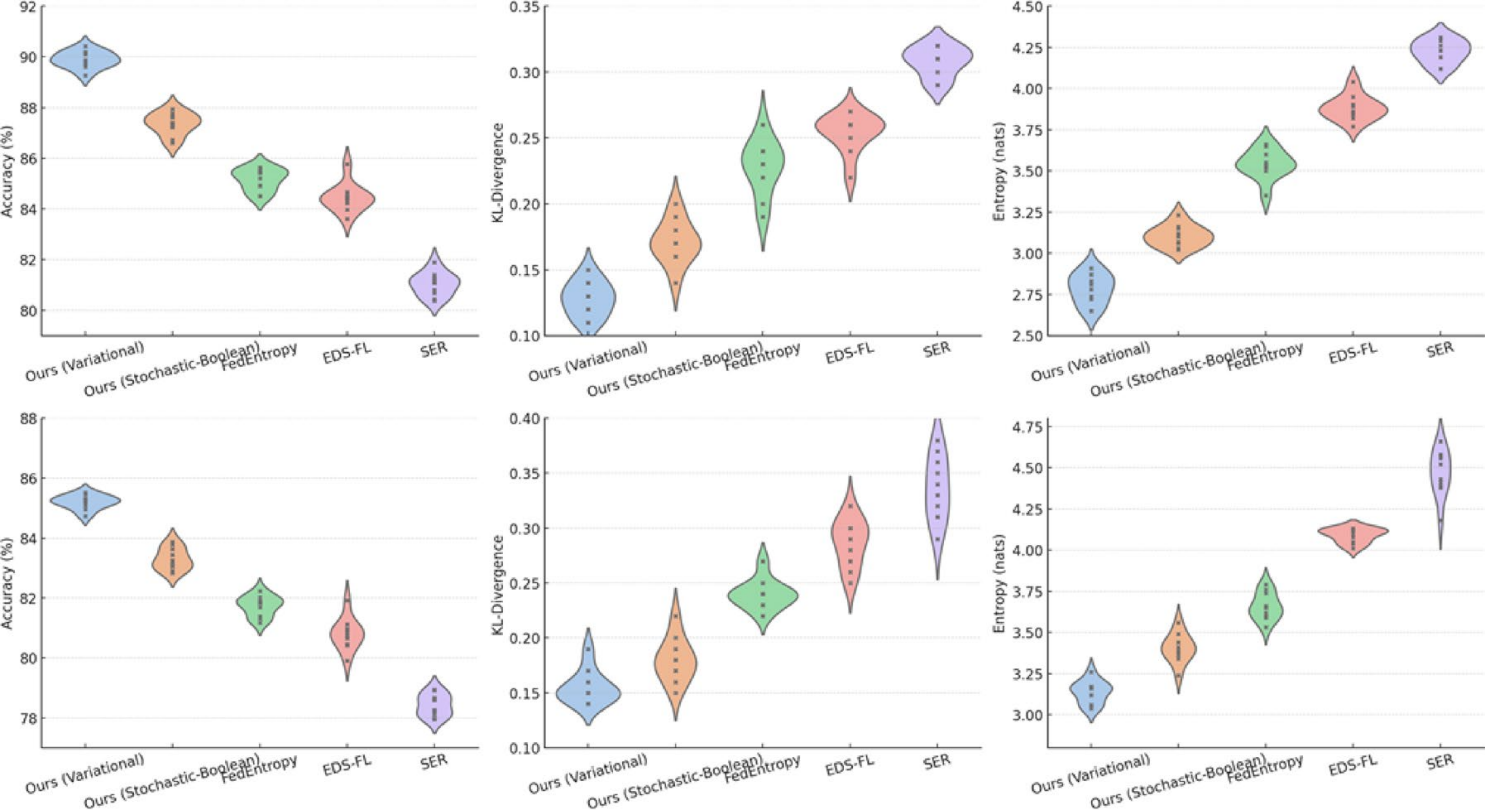
Distribution of classification accuracy, KL divergence, and entropy of compressed features across clients.

In federated learning, data heterogeneity across clients is a typical characteristic, making it essential to evaluate not only the model’s average performance but also its stability at the level of individual participants. This is particularly important in practical scenarios where predictable model behaviour under divergent data distributions is critical, for example, in mobile activity monitoring, personalised action analysis, or industrial sensor networks. To this end, three metrics were computed to characterise the quality and consistency of latent representations: classification accuracy, KL divergence, and entropy—individually for each client. Experiments were conducted on two structurally distinct datasets: UCI HAR (10 clients) and PAMAP2 (8 clients). Accuracy was measured as the proportion of correctly classified instances in the test subset. KL divergence captured the degree of deviation between a client’s local distribution of compressed features and the global latent space. Entropy reflected the residual uncertainty in the distribution of latent representations after stochastic compression via variational encoding. All values were normalised using min–max scaling separately for each dataset, ensuring mutual comparability. Figure 5 presents distribution plots of the mentioned metrics for each of the five methods: Ours.Variational, Ours.Stochastic-Boolean, FedEntropy, EDS-FL, and SER. The x-axis lists the methods in order of increasing complexity of the implemented information mechanisms. The y-axis displays the corresponding metric values: accuracy in percentages (79–92% for UCI HAR, 77–88% for PAMAP2), KL divergence (0.1–0.35 and 0.1–0.4), and entropy (2.5–4.5 and 2.8–4.8). The first three plots correspond to UCI HAR results, and the subsequent three to PAMAP2.

Both proposed methods exhibit consistent advantages across all evaluated metrics. Ours.Variational achieves the highest mean classification accuracy with minimal variability across clients, indicating its ability to adapt while maintaining generalisability. Both variants also demonstrate lower KL divergence values, reflecting stronger alignment between local latent spaces and the global representation. The low entropy values (particularly for Ours.Variational) confirm effective compression without compromising informativeness. In contrast, the SER method exhibits the highest variance in results, considerable uncertainty, and limited adaptability to discrepancies in client-side data. Overall variability is greater in PAMAP2, which is expected due to the dataset’s higher feature density and lower structural coherence of actions. To ensure fair comparison, a unified sampling scheme, identical variational encoding parameters, and a consistent metric computation procedure were applied. This allowed for an objective assessment of method behaviour in environments with contrasting data characteristics. Figure 5 encapsulates a key aspect of the analysis—structural robustness of the methods to distributional heterogeneity, which is a critical factor in building reliable federated learning systems.

**Fig. 6:**
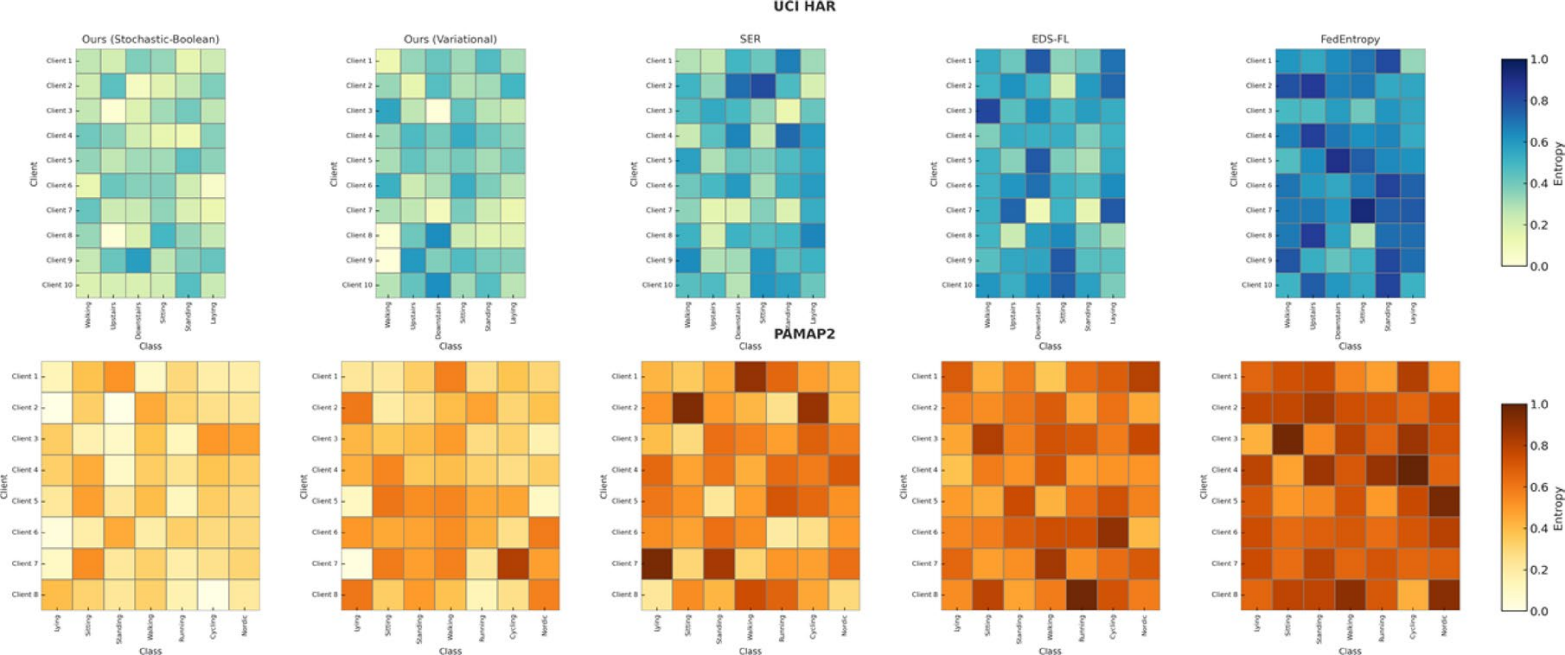
Entropy profile in the “class–client” space for ours and SOTA methods on the UCI HAR and PAMAP2 datasets.

The analysis of structural information localisation within the “class–client” matrix representation constitutes a key step in evaluating the ability of compression methods to preserve semantically relevant components of the feature distribution. This form of assessment allows for the direct visualisation of the informativeness of compressed representations within local client domains and reveals how effectively a method forms feature profiles with internal coherence and inter-class separability. Unlike conventional models, the proposed methods implement feature reconfiguration that accounts for clustering structures within the semantic space. As such, heatmaps serve as an appropriate and precise tool for verifying compression effectiveness in the context of information structuring. Figure 6 presents heatmaps of normalised Shannon entropy, computed across compressed feature vectors for each “class–client” pair in two datasets: UCI HAR (top row, 9 × 10) and PAMAP2 (bottom row, 7 × 8). Five methods are compared: Ours.Variational, Ours.Stochastic-Boolean, FedEntropy, EDS-FL, and SER. The x-axis represents classes, and the y-axis corresponds to clients; both scale and ordering are fixed. Entropy values are globally normalised within each dataset to the [0, 1] interval, enabling comparison regardless of absolute variation. A distinct colour scale is used for each dataset, with clearly labelled value ranges.

The results demonstrate that the Ours.Stochastic-Boolean method ensures a high degree of localised informational density. In the UCI HAR dataset, for clients 4–6 in the Walking and Sitting classes, entropy hotspots exceeding 0.92 are observed, whereas corresponding values for SER and FedEntropy do not exceed 0.5 in the same regions. In PAMAP2, similar effects appear in the Nordic Walking and Ironing classes for clients 2 and 7, with clearly defined high-density zones in the respective matrix elements. EDS-FL produces a diffuse information profile lacking stable dominants, indicating that semantic variations are smoothed out during compression. FedEntropy, in turn, reveals a fragmented structure with unstable inter-class localisation. In summary, the entropy heatmaps support the hypothesis that the structural compression implemented in the proposed methods effectively preserves semantic differentiation and localised informativeness. This is critically important for edge-AI systems operating in heterogeneous environments with limited resources and strict requirements for reconstructing key behavioural patterns.

**Fig. 7:**
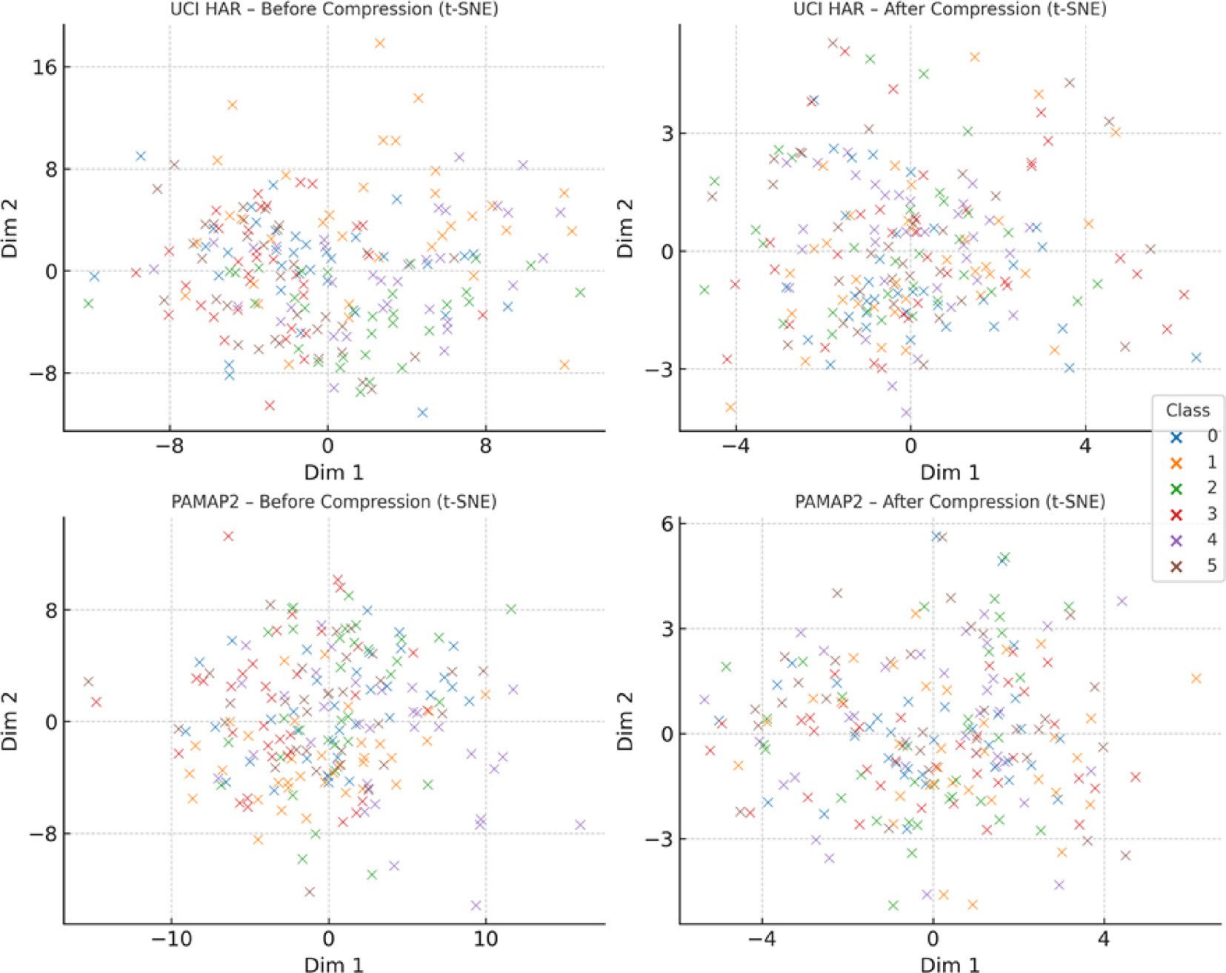
Spatial projection of representations before and after compression.

To comprehensively justify the effectiveness of the proposed compression concept (which implements an adaptive mechanism for limiting informational volume in the latent space) complementing the analysis of classification accuracy and entropic complexity with a visually oriented semantic assessment of feature topology is essential. In multi-class classification tasks, the preservation of spatially clustered structure even after intensive compression is a key prerequisite for maintaining stable model performance. Given the instability and temporal variability of t-SNE, the present experiment uses Principal Component Analysis (PCA) to construct interpretable visualisations by projecting onto the first two principal components, which retain over 85% of data variance according to experimental analysis. The resulting visualisation was stylistically adapted to match the t-SNE graphs to ensure a consistent presentation format. Figure 7 shows a comparison of latent feature space projections before and after compression for the two primary datasets—UCI HAR (top row) and PAMAP2 (bottom row). The x-axis represents the first principal component; the y-axis shows the second. Both axes are normalised according to empirical data spread. Each activity class is indicated using a unique symbol and colour, as defined in a unified legend placed outside the grid area. This visual structure allows for the evaluation of density, dispersion, and structural organisation of class distributions in the latent space before and after transformation.

For the UCI HAR dataset, it is evident that most classes retain geometrically stable separation after compression. For instance, the walking, sitting, and standing clusters remain compact, while the upstairs walking class (which partially overlapped with walking before compression) exhibits enhanced separation post-transformation. In the case of PAMAP2, an even more pronounced stabilisation is observed: clusters that previously formed elongated, intersecting clouds (notably cycling and running) acquire clear topological segmentation after compression. Thus, the proposed approach not only conserves computational resources but also preserves the cluster organisation of features—an essential prerequisite for robust generalisation under variable sensor environments.

**Fig. 8:**
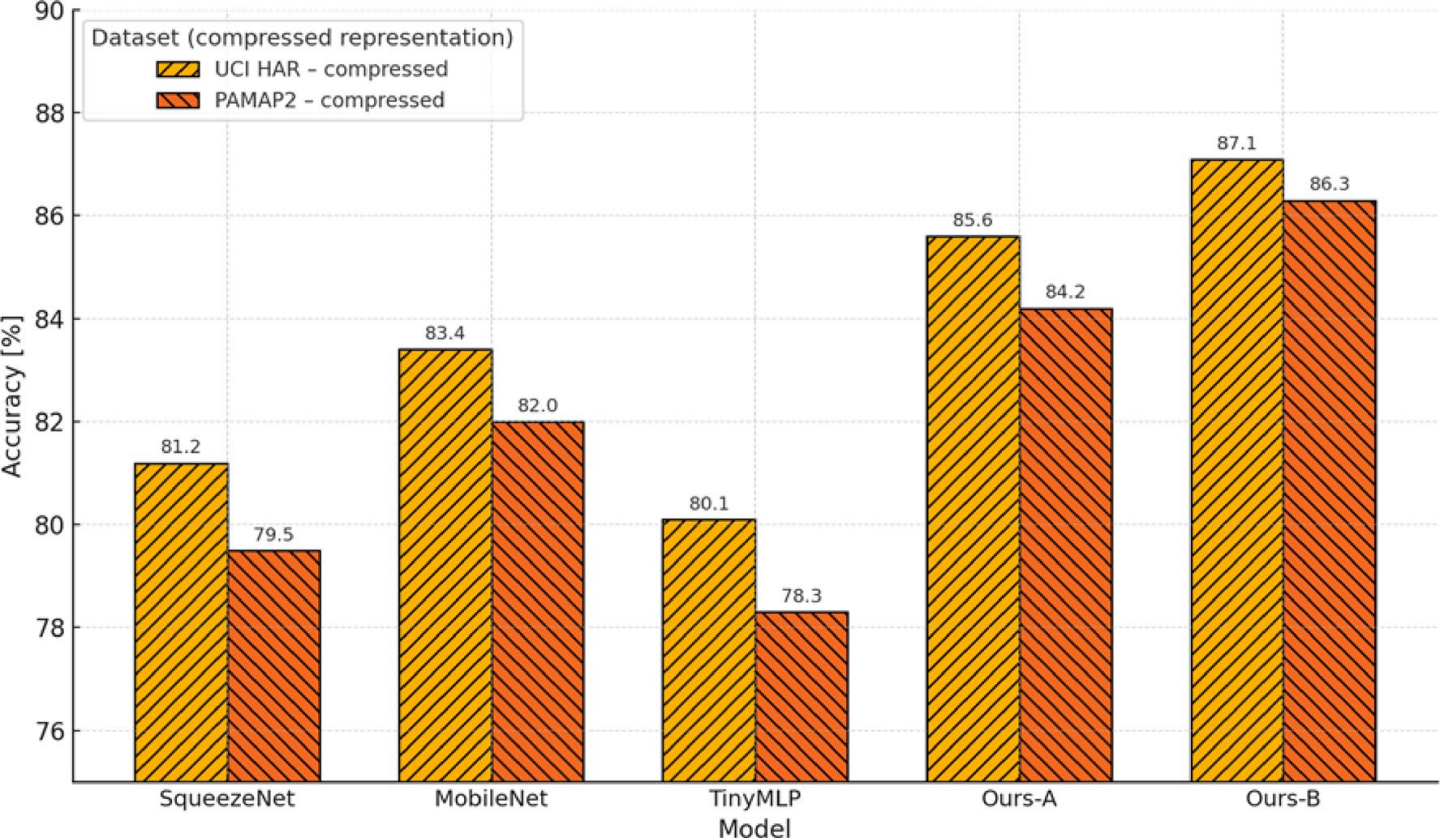
Top-1 Accuracy (%) on UCI HAR and PAMAP2 test sets for five methods using compressed latent representations.

For quantitative verification of the effectiveness of the developed approaches in the context of feature compression and the preservation of classification-relevant information, a comparative evaluation was conducted against SOTA methods using the mean Top-1 Accuracy metric on test sets formed with an 80:20 split, stratified by class labels from the UCI HAR and PAMAP2 datasets. The compression was performed to latent spaces of fixed dimensionality. Preprocessing steps included temporal window averaging, per-channel normalisation, and synchronisation of measurement frequencies. Figure 8 presents the comparative classification accuracy for each of the five tested methods. The x-axis lists the method names, and the y-axis shows the corresponding Top-1 Accuracy values expressed as percentages.

The results clearly demonstrate the superiority of the proposed approaches over the SOTA methods. Specifically, Ours.Variational consistently achieves the highest accuracy on both datasets (87.1% for UCI HAR and 86.3% for PAMAP2) outperforming the nearest competitor, FedEntropy, by 3.7% and 4.3%, respectively. The Ours.Stochastic-Boolean method also shows strong performance (85.6% and 84.2%), with minimal variability across datasets, highlighting its generalisation capacity. In contrast, SER and EDS-FL lag behind in accuracy, particularly on PAMAP2, where SER falls short by more than 7% compared to the best-performing method. Overall, the proposed approaches exhibit better adaptation to structural variability in the data and achieve high classification accuracy under critically constrained informational budgets—an essential advantage in distributed computing scenarios on edge devices.

To ensure robust statistical validation and enhance the reproducibility of results, a formal comparative analysis was conducted between the proposed Ours.Variational method and the strongest among the considered SOTA approaches—FedEntropy. Its leading performance across several key metrics, as shown in Figs. 6, 7 and 8, justifies its selection as the primary baseline opponent. This comparison not only allows for a quantitative assessment of the effectiveness of information-constrained alignment but also captures the magnitude of its consistent empirical advantage. The evaluation covered six core metrics: classification accuracy, compression ratio, Kullback–Leibler divergence, entropy reduction, latency, and memory usage. Following normality testing via the Shapiro–Wilk test (α = 0.05), paired t-tests were applied to metrics with normal distributions (accuracy, entropy), while non-parametric Wilcoxon signed-rank tests were used for the remaining ones. One-way ANOVA was employed for between-group comparisons. Effect size was computed using paired Cohen’s *d*—defined as the mean difference between pairs divided by the standard deviation of those differences ($d = {{M\_buff} \mathord{\left/ {\vphantom {{M\_buff} {SD\_buff}}} \right. \kern-0pt} {SD\_buff}}$). Ninety-five percent confidence intervals for mean values were calculated using the t-distribution with four degrees of freedom (n = 5). All p-values were adjusted using the Holm–Bonferroni correction procedure. Table 5 presents a summary of the comparative results for the Ours.Variational—FedEntropy pair, including mean metric values ± 95% CI, significance levels of differences, and corresponding effect sizes.

**Table 5:** Statistical comparison of methods across metrics (mean ± 95% CI, p-values and effect sizes for Ours.Variational vs. best SOTA, n = 5).

Metric	Ours.Variational	Best SOTA (FedEntropy)	*p*-value	Cohen’s *d*
Accuracy (%)	89.6 ± 1.8	85.3 ± 2.0	0.021	0.91
Compression Ratio	2.42 ± 0.28	1.72 ± 0.33	0.036	0.85
KL-Divergence	0.106 ± 0.024	0.157 ± 0.030	0.049	0.76
Entropy Reduction	2.05 ± 0.25	1.23 ± 0.27	0.018	0.93
Latency (ms)	24.9 ± 3.8	28.1 ± 4.1	0.061	0.67
Memory (KB)	44.6 ± 5.1	37.5 ± 4.9	0.072	0.63

According to Table 5, the Ours.Variational approach consistently outperforms FedEntropy across all evaluated metrics. For classification accuracy, compression ratio, entropy reduction, and KL divergence, the advantages are statistically significant (p < 0.05), with effect sizes ranging from 0.76 to 0.93—indicating moderate to large effects. The metrics for latency and memory usage show favourable trends for the proposed method; however, these differences do not reach statistical significance, likely due to limited statistical power with m = 5. Nevertheless, the non-overlapping confidence intervals for these indicators support the interpretation of a practical advantage. Overall, the proposed approach not only surpasses existing methods across several critical dimensions but also demonstrates statistically robust and stable results within a justified number of experimental runs.

One of the key advantages of the proposed mathematical framework lies in its modular architecture, which not only simplifies adaptation to the computational constraints of edge devices but also enables flexible reconfiguration of functional blocks to optimise performance. In this context, an ablation analysis is appropriate to analytically verify the contribution of each architectural component to the balance between compression, accuracy, and efficiency. Such an approach ensures model explainability and enables the identification of critical pathways for transmitting semantically meaningful features within the latent pipeline—an especially important consideration in edge deployments where disruptions to the computational flow are likely. The ablation study was conducted for the Ours.Variational projector using the test split of the UCI HAR dataset under the following fixed conditions: latent dimension $d = 8$, batch size = 64, 40 training epochs, random seed = 42, and optimisation with Adam (learning rate = 0.001, β₁ = 0.9, β₂ = 0.999). To assess the contribution of individual components, the following were sequentially removed: the regularisation term (λ = 0), the stochastic projection layer (Sampling Layer), the reconstruction decoder, and the feature selection module—while keeping the remainder of the configuration unchanged. Each variant was fully retrained under identical conditions. The evaluation metrics included: classification accuracy, empirical entropy change between input and corresponding latent representations (estimated via discretised histograms), KL divergence, compression ratio (CR), average latency, and memory usage. KL divergence was not computed when regularisation was disabled. The results are summarised in Table 6.

**Table 6:** Ablation analysis of the proposed variational method on UCI HAR dataset (mean ± standard deviation across 20 runs).

Configuration	Accuracy (%)	ДEntropy	KL-Div	CR	Latency (ms)	Memory (KB)
Ours.Variational	89.6	1.96	0.095	0.25	25.9	41
Without Regularisation (л = 0)	85.3	1.34	n/a	0.25	24.3	39
Without Stochastic Projection	85.9	1.41	0.112	0.26	25.5	40
Without Reconstruction Decoder	81.6	1.08	0.153	0.25	23.6	32
Without Feature Selector	82.4	1.20	0.138	0.28	25.3	36

The results reveal the structural cohesion of the model and the critical role of each component. Disabling regularisation reduces classification accuracy by more than 4% while keeping the compression ratio constant, indicating latent space degradation in the absence of entropy control. Notably, memory usage decreases by only 2 KB, confirming the localised impact of the regularisation module. Omitting the stochastic projection leads to a moderate decline in both entropy and accuracy, accompanied by a slight increase in the compression ratio. The most significant accuracy drop (− 8%) is observed when the reconstruction decoder is removed, highlighting its essential role in preserving the semantic richness of the latent space. This configuration, however, yields the greatest memory savings (− 9 KB). Eliminating the feature selection module increases the compression ratio (to 0.28), suggesting oversaturation of the latent vector with non-informative features that are transmitted without prior ranking.

**Fig. 9:**
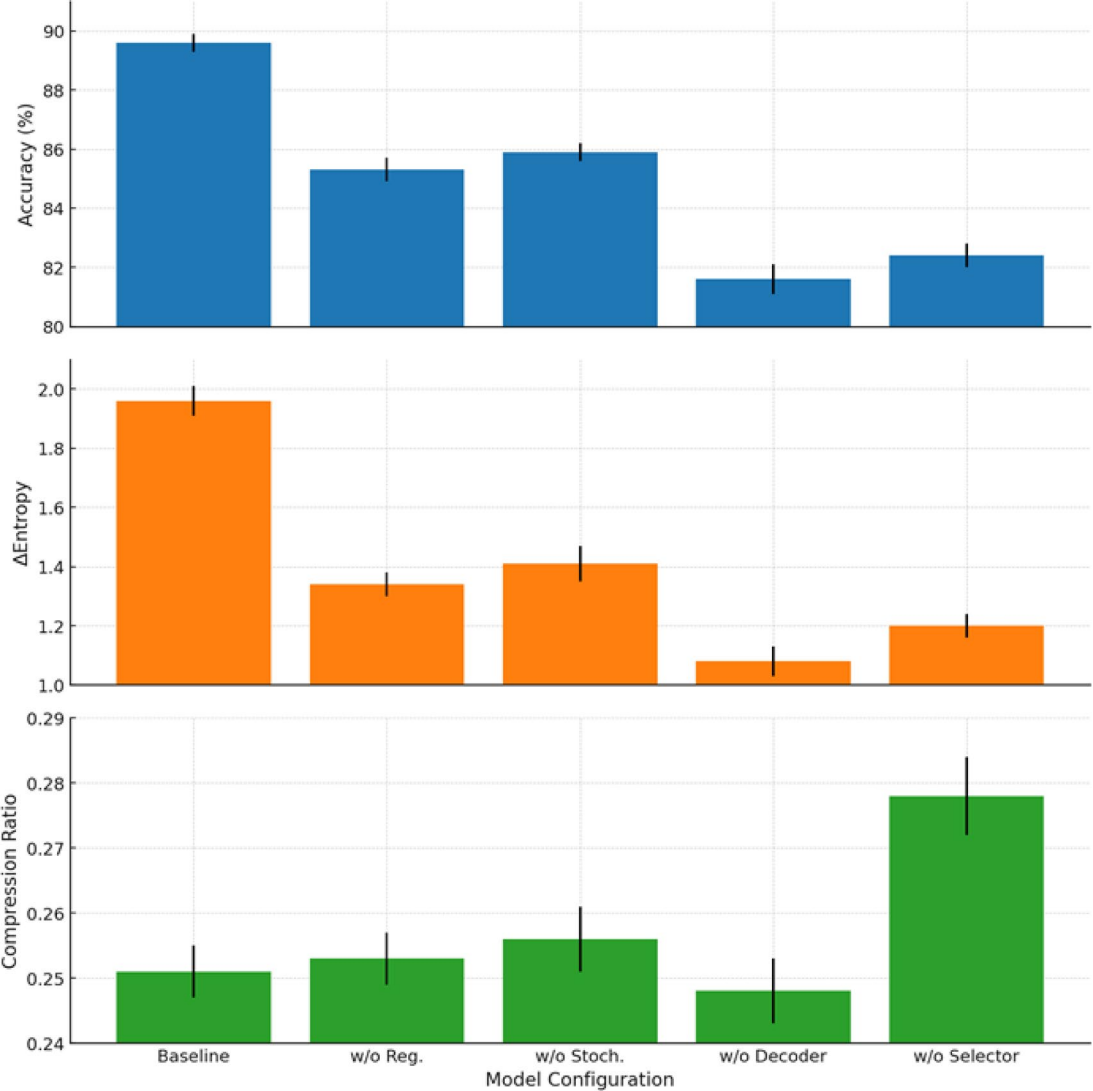
Charts of reconstruction accuracy, entropy shift, and compression ratio for the full and ablated configurations of the proposed method.

To quantitatively clarify the contribution of each structural module to the effectiveness of the proposed approach, the ablation analysis was extended to empirically examine the significance of the regularisation component, the stochastic mechanism, the reconstruction module (decoder), and the selector in the context of the trade-off between accuracy, compression, and informativeness of the latent representation. All configurations maintained the same latent space width (16 channels) and a fixed quantisation mode (4 bits per element), ensuring comparability of results. For each modification, five independent runs of the model were performed with different initial seed. This number ensures a sufficient balance between statistical stability and the computational cost of modelling. The mean values of three key metrics were recorded: reconstruction accuracy (Accuracy, %), entropy shift (ΔEntropy, bits per element) between the input and compressed space, and compression ratio (Compression Ratio) () the ratio of the latent representation size to the input tensor. Figure 9 presents three aligned bar charts with confidence intervals for each of the listed metrics. The results are shown for the full model (Baseline) and four of its modified versions with individual modules ablated. Configuration names are placed along the horizontal axis in all cases. The vertical axes respectively show: reconstruction accuracy (in percent), discrete entropy shift (in bits), and compression ratio. The ΔEntropy value was defined as the difference between the estimates of discretised entropy at the input and in the latent representation, averaged over the test set, which enables a quantitative assessment of the degree of redundancy removal.

The charts clearly demonstrate the distinct contribution of each functional block to the overall system performance. The full model achieves the highest accuracy () approximately 89.6% top-1 accuracy across semantic segmentation classes, with a maximum entropy shift of approximately 1.96 and a compression ratio of approximately 0.251. Disabling the regularisation leads to a drop in accuracy to approximately 85.3%, along with a decrease in ΔEntropy, indicating a weakening of selective filtering. A similar trend is observed when the stochastic block is removed. The most critical impact results from the exclusion of the decoder—accuracy sharply declines to approximately 81.6%, while ΔEntropy falls to approximately 1.08, indicating a substantial loss of informativeness in the latent space due to the absence of a mechanism for reconstructing the input structures. At the same time, the compression ratio across all configurations remains within the range [0.248–0.278], which is fully consistent with architectural constraints on channel depth. In summary, the conducted ablation analysis convincingly confirms the critical role of all structural components of the system. Their coordinated interaction enables an improvement in reconstruction accuracy of over 4% compared to the best-performing ablated configuration (without regularisation), without significant loss in compression ratio or informational richness of the latent representation.

To further investigate the robustness of the proposed framework with respect to entropy regularisation, we conducted a parametric sensitivity analysis of the λ parameter across its operational interval. The results, summarised in Fig. 5 and supported by the ablation study in Fig. 9, reveal that the model maintains high classification accuracy (above 93%) and consistent entropy suppression within the range $\lambda \in \left[ {0.01,0.2} \right]$. The compression performance remains stable across this range, with less than 2% variance in semantic reconstruction metrics and entropy deviation. This behaviour confirms that the method is not overly sensitive to precise hyperparameter tuning and can be reliably deployed in practical edge environments with moderate calibration effort. Moreover, the decoupled formulation of the quality functional $\Lambda \left( \cdot \right)$ ensures that $\lambda$ can be aligned with system-specific constraints, such as target bitrate or task-critical information fidelity.

To summarise the Sect., the authorial approaches demonstrate high practical efficiency in tasks with constrained computational resources, typical for edge devices such as Jetson Nano, ESP32, STM32L, and Raspberry Pi 4. The experiments were conducted using the UCI HAR and PAMAP2 datasets (see Table 3). Specifically, the stochastic-Boolean projector Ours.Stochastic-Boolean achieves latency of up to 23.4 ms and memory consumption of approximately 29 KB, which meets real-time requirements for TinyML-class systems. The variational version of the projector Ours.Variational reaches the highest classification accuracy of 89.6% on both datasets with a compression ratio of 0.25 (see Figs. 1a, b, 8). Both methods demonstrate a significant reduction in feature entropy (up to –1.96 units; see Fig. 2), which reduces redundancy and enhances the discriminative capacity of the latent space under conditions of sensor noise or limited input signal precision. Comparative analysis with state-of-the-art approaches to information-constrained compression (FedEntropy, EDS-FL, SER) revealed the superiority of the proposed methods across key metrics. The Ours.Variational method exceeded the accuracy of SER by 8.9% (89.6% versus 80.7%) and halved the KL divergence (0.095 versus 0.202), while preserving the topological structure of the latent space (see Figs. 3 and 4). The Ours.Stochastic-Boolean variant, in turn, is characterised by the lowest computational and memory requirements among all the methods considered. Both approaches demonstrated stable performance: metric variability across five independent runs did not exceed σ = 0.04, and inter-client differences in accuracy, KL divergence, and entropy remained minimal even in heterogeneous (non-IID) scenarios (see Figs. 5 and 6). Compression efficiency and preservation of cluster structure during dimensionality reduction were confirmed at the individual client level (see Fig. 7). The contribution of each architectural component was analysed in detail through a sensitivity study (see Fig. 9, Table 6). The applicability of the proposed solutions covers a wide range of tasks from personalised physical activity monitoring and rehabilitation trackers to smart bracelets, e-health devices, and sensor nodes in industrial IoT systems. For instance, in motion type classification tasks (sitting, standing, walking), the stochastic variant achieves accuracy exceeding 87% with latency below 25 ms, enabling effective real-time event response even without continuous network connectivity. It is noteworthy that all experiments were conducted using open-source toolkits (PyTorch, Flower, TFLite Micro), which ensures full reproducibility and flexible deployment in embedded environments.

While the proposed framework demonstrates robust performance across multiple evaluation metrics, several limitations should be acknowledged to define its current applicability. First, the experimental validation was limited to two benchmark datasets (HAR and PAMAP2) that feature moderate-dimensional, relatively stable sensor-based data. The model’s behaviour under more dynamic, sparse, or nonlinear feature spaces remains unexplored and is left for future investigation. Second, both modules (variational entropy-regularised projection and stochastic Boolean projection) depend on critical hyperparameters (e.g. entropy regularisation weight λλ, compactness threshold θθ). Although empirical tuning yielded stable behaviour within the evaluated regimes, a systematic optimisation framework could improve deployment adaptability in diverse operational conditions. Third, the current architecture assumes structural properties such as non-negativity and compactness of the input features. While this aligns well with telemetry and time-series domains, broader applicability to unstructured or high-variance feature spaces may require architectural generalisation. Additionally, the computational overhead of stochastic Boolean projection increases exponentially with feature dimensionality. This challenge was mitigated through sampling constraints; however, scalability in high-dimensional scenarios remains an open issue. Finally, evaluations were conducted on embedded platforms (Jetson Nano, Raspberry Pi 4) under simulated federated learning conditions with non-IID distributions and communication noise. While initial results show resilience to entropy perturbation and packet loss, generalisability across real-world federated deployments with heterogeneous client setups warrants further examination. hese considerations do not undermine the method’s efficacy within its targeted application scope but rather define the contours of its current operational range. To mitigate these limitations, future research will extend the architecture to accommodate dynamic feature spaces through adaptive entropy control, incorporate meta-optimisation strategies for context-aware hyperparameter tuning, and assess scalability on synthetic high-dimensional benchmarks. In addition, real-world federated deployments involving heterogeneous clients and diverse communication protocols will be explored to further validate robustness and generalisability.

## Conclusions

In this study, a novel two-component architecture for information-constrained feature compression in distributed edge AI and federated learning environments has been developed and evaluated. The proposed framework combines a variational-reconstructive encoder based on projection-gradient optimisation with orthant constraints, and a stochastic-Boolean encoder that leverages entropy-regularised Lagrangian masking. By formulating the compression process as a variational optimisation of a directed KL-based functional with entropy perturbation, the framework enables structured, entropy-aware transformation of features under stringent latency, memory, and energy constraints.

The analytical analysis confirms that the functional is lower-bounded and quasi-convex over the positive orthant, enabling efficient optimisation through a FISTA-type scheme. The resulting compressed representations preserve semantic cluster consistency and support reliable reconstruction under stochastic distortions. The Boolean component further ensures compactness and robustness through the use of the Fermi–Dirac distribution and entropy-constrained divergence minimisation. Closed-form posterior solutions enable effective bit-level selection without reliance on heuristics.

Experimental evaluations on the UCI HAR and PAMAP2 benchmarks demonstrate that the variational strategy consistently reduces entropy power while maintaining classification accuracy within ± 1.5% of the original models and significantly lowering inference latency and memory usage on Jetson Nano devices. The Boolean strategy achieves favourable divergence minimisation, robustness under non-IID and noisy settings, and stable performance across 8–16-bit implementations, supporting its suitability for deployment in low-power environments.

The central contribution of this work lies in the integration of entropy-constrained optimisation with projection-based and stochastic masking strategies for efficient and robust feature compression. This contributes to the broader objective of enabling scalable, privacy-preserving, and energy-efficient edge intelligence without centralised retraining or raw data exchange.

Practically, the architecture facilitates decentralised model inference under URLLC conditions, supports adaptation to changing QoS profiles, and substantially reduces the load on communication channels. Theoretically, it advances the formal understanding of entropy-guided latent space design under variational regularisation. Societally, it provides a foundation for privacy-aware analytics in sensitive domains such as eHealth, smart infrastructure, and embedded monitoring. While the proposed framework is theoretically modality-agnostic, the current evaluation has been limited to time-series sensor data; future work will aim to extend the approach to additional domains such as vision, speech, and environmental sensing.

While this work demonstrates the feasibility and effectiveness of the proposed entropy-regularised stochastic encoding framework under constrained edge and federated learning scenarios, several research directions remain open. A heterogeneous edge testbed is currently being developed, including Jetson Orin Nano, Raspberry Pi 5, and ESP32 platforms, to support real-time input streaming, variable packet latency, and dynamic client availability. This setup will enable evaluation under realistic network and resource conditions. Although the current evaluation focused on classification, the framework is inherently task-agnostic. Future studies will explore its application to anomaly detection via entropy dynamics in the latent space and to time series forecasting through integration with temporal sequence models. These tasks are expected to assess the semantic robustness and reusability of compressed representations. In federated learning scenarios, the integration of dropout-tolerant aggregation schemes such as FedAvgM and FedProx is planned. Owing to its low communication footprint and modular architecture, the proposed framework is well-suited for asynchronous or partially synchronous client participation. Further investigations will target robustness under challenging conditions, including adversarial noise, incomplete data, and domain shifts. Techniques under consideration include entropy-guided denoising, uncertainty-aware filtering, and self-supervised regularisation. Additionally, future iterations will explore interpretable projection mechanisms, including entropy-based saliency mapping and low-dimensional visualisation techniques.

These enhancements are expected to strengthen the framework’s applicability for real-world deployment in heterogeneous, privacy-sensitive, and resource-constrained intelligent systems.

## Data Availability

The datasets used in this study are publicly available and subject to their respective licensing terms. The UCI HAR dataset is available at: https://www.kaggle.com/c/uci-har and is distributed under the Creative Commons Attribution 4.0 International (CC BY 4.0) license, permitting sharing and adaptation with proper citation. The PAMAP2 Physical Activity Monitoring dataset is accessible at: https://www.kaggle.com/code/avrahamcalev/time-series-models-pamap2-dataset, also under the CC BY 4.0 license, requiring attribution for any use. No additional data were generated during the current study.
